# Supplementary material for: A Study on Synthesis and Upscaling of 2′-O-AECM-5-methyl Pyrimidine Phosphoramidites for Oligonucleotide Synthesis
Source: Molecules. 2021 Nov 17;26(22):6927. doi: 10.3390/molecules26226927 (PMC8619030; doi:10.3390/molecules26226927)
Supplement: Supplementary file 1 [file molecules-26-06927-s001.zip › molecules-1444195-supplementary.pdf]

Supporting Information

A Study on Synthesis and Upscaling of 2'-O-AECM-5-methyl Pyrimidine Phosphoramidites for Oligonucleotide Synthesis

Kristina Karalė [1,2,4], Martin Bollmark[2], Rouven Stulz[3], Dmytro Honcharenko [1], Ulf Tedebark[2] and Roger Strömberg [1,*]

[1] Department of Biosciences and Nutrition, Karolinska Institutet, Neo, 141 83 Huddinge, Stockholm, Sweden; kristina.druceikaite@ki.se (K.K.), dmytro.honcharenko@ki.se (D.H), roger.stromberg@ki.se (R.Sg)

[2] RISE, Department Chemical Process and Pharmaceutical Development, Forskargatan 18, SE-15136 Södertälje, Sweden; kristina.druceikaite@ri.se (K.K.), martin.bollmark@ri.se (M.B.), ulf.tedebark@ri.se (U.T.)

[3] Oligonucleotide Discovery, Discovery Sciences, BioPharmaceuticals R&D, AstraZeneca, Gothenburg, Sweden; rouven.stulz@astrazeneca.com (R.Sz).

[4] née - Druceikaite

* Correspondence: roger.stromberg@ki.se; Tel.: +46852481024

Contents

Tables S1-S2 Screen for different conditions for 2´OH alkylation of compound **3**……………………………………………S4

Table S3 Screen for different conditions for N4 acetylation of compound **13**………………………………………………S5

Figure S1 HPLC profile of crude compound **2** in 50 g scale synthesis……………………………………………………..S6

Figures S2-S3 HPLC profiles of screen for different conditions for 2´OH alkylation of compound **3**……….....................S7-S8

Figures S4-S15 ^{1}H NMR, ^{13}C NMR, ^{19}F NMR and ^{31}P NMR spectra for MeU intermediates..……………….…………...S9-S20

Figures S16-S33 ^{1}H NMR, ^{13}C NMR, ^{19}F NMR and ^{31}P NMR spectra for MeC intermediates………………………………S21-S38

Figures S34-S37 HPLC profiles of MeU intermediates………………...…………………………………………........S39-S42

Figures S38-S45 HPLC profiles of MeC intermediates………………...……………………………………………….S43-S50

Figures S46-S57 ESI-TOF mass spectra for MeU and MeC intermediates…….……………………………………...S51-S62

*Table S1. Screen for different conditions of MeU 2´alkylation (compound **3**).*

No.	Methyl 2-bromoacetate	Solvent, 5 vol	K_2CO_3	PTC catalyst, 0.2 equiv.	HPLC area%, conver., 40°C, 1h	HPLC area%, conver., 40°C, 4.5 h	HPLC area%, conver., RT, overnight
1.	2 equiv.	MeCN	2 equiv.	TBABr	26%	71%	89%
2.	4 equiv.	MeCN	2 equiv.	TBABr	36%	78%	85%
3.	2 equiv.	MeCN	4 equiv.	TBABr	42%	81%	84%
4.	4 equiv.	MeCN	4 equiv.	TBABr	46%	80%	88%

*Table S2. Screen for different conditions of MeU 2´alkylation (compound **3**).*

No.	Methyl 2-bromoacetate	Solvent, 5 vol	Base, 4 eq	PTC agent, 0.2 equiv.	HPLC area%, conver., RT, over weekend
1.	2 equiv.	Heptane	K_2CO_3	TBABr	96%
2.	2 equiv.	MeCN	K_2CO_3	TBABr	85%
3.	2 equiv.	Heptane	K_3PO_4	TBABr	96%
4.	2 equiv.	MeCN	K_3PO_4	TBABr	85%

*Table S3. Screen for different conditions for N4 acetylation MeC (compound **13**).*

No.	Solvent, 5 vol	A	B	C	D	E	F	G
1.	MeOH	14%	7%*	0%	11%	0%	0%	0%
2.	DCM	55%	82%	84%	92%	92%	90%	80%
3.	Toluene	43%	79%	84%	89%	88%	86%	81%
4.	DMF	67%	86%	83%	86%	82%	80%	83%
5.	MeCN	59%	87%	85%	87%	84%	83%	84%

*degradation

A= HPLC area%, conver., RT, 4.5 h
B= HPLC area%, conver., RT, overnight, +0.5 equiv. Ac_2O
C= HPLC area%, conver., +0.5 equiv. Ac_2O, over weekend
D= HPLC area%, conver., +0.5 equiv. Ac_2O, over weekend, additional 0.5 equiv. Ac_2O, 1h
E= HPLC area%, conver., +0.5 equiv. Ac_2O, over weekend +0.5 equiv. TEA, 1 h
F= HPLC area%, conver., +0.5 equiv. Ac_2O, over weekend, additional 0.5 equiv. Ac_2O, 1h, then 0.5 equiv. TEA
G= HPLC area%, conver., +0.5 equiv. Ac_2O, over weekend, additional 0.5 equiv. Ac_2O, 1h, then 0.5 equiv. Ac_2O

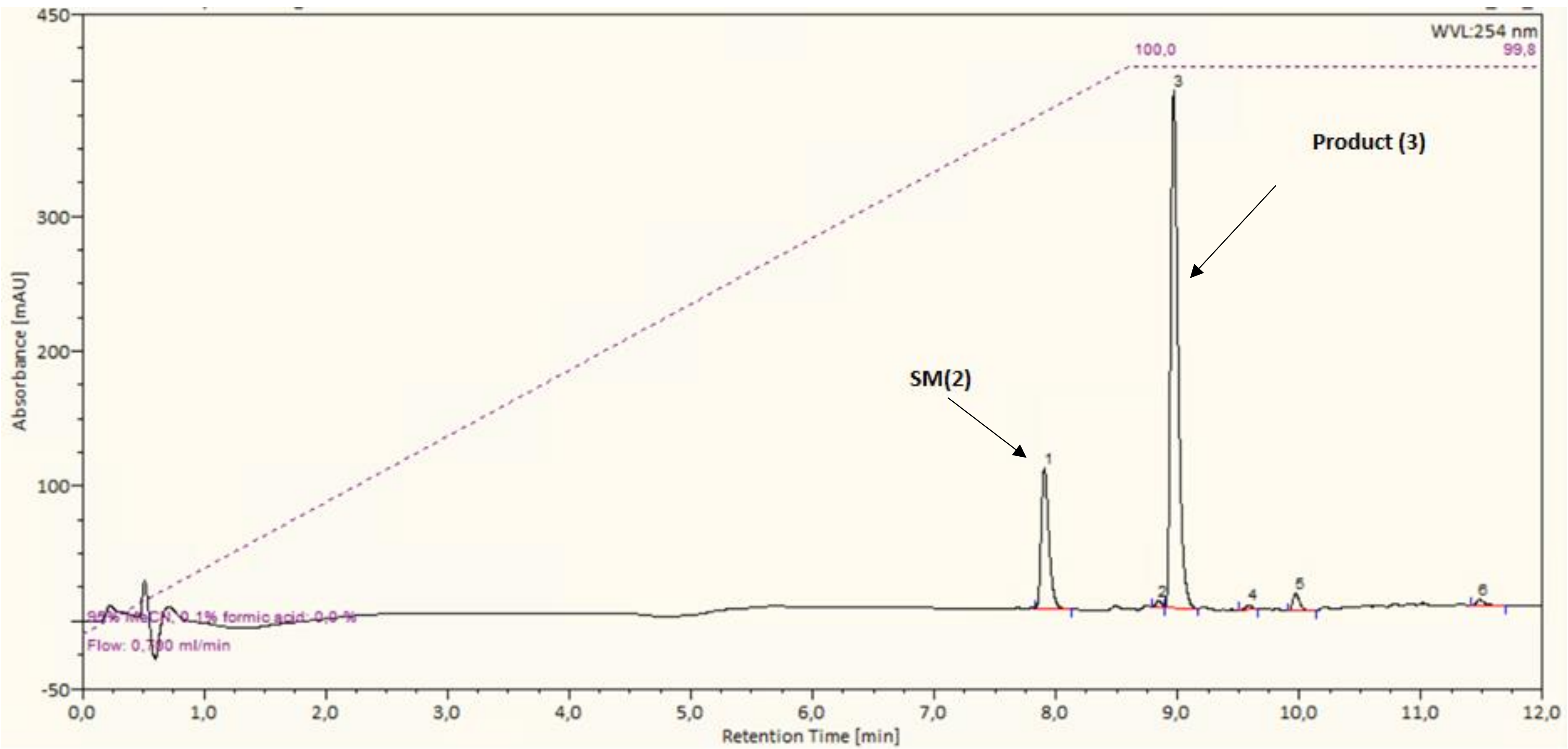

Figure S1.HPLC profile of crude N³ alkylation reaction on MeU in 50 g scale after 5.5 h.

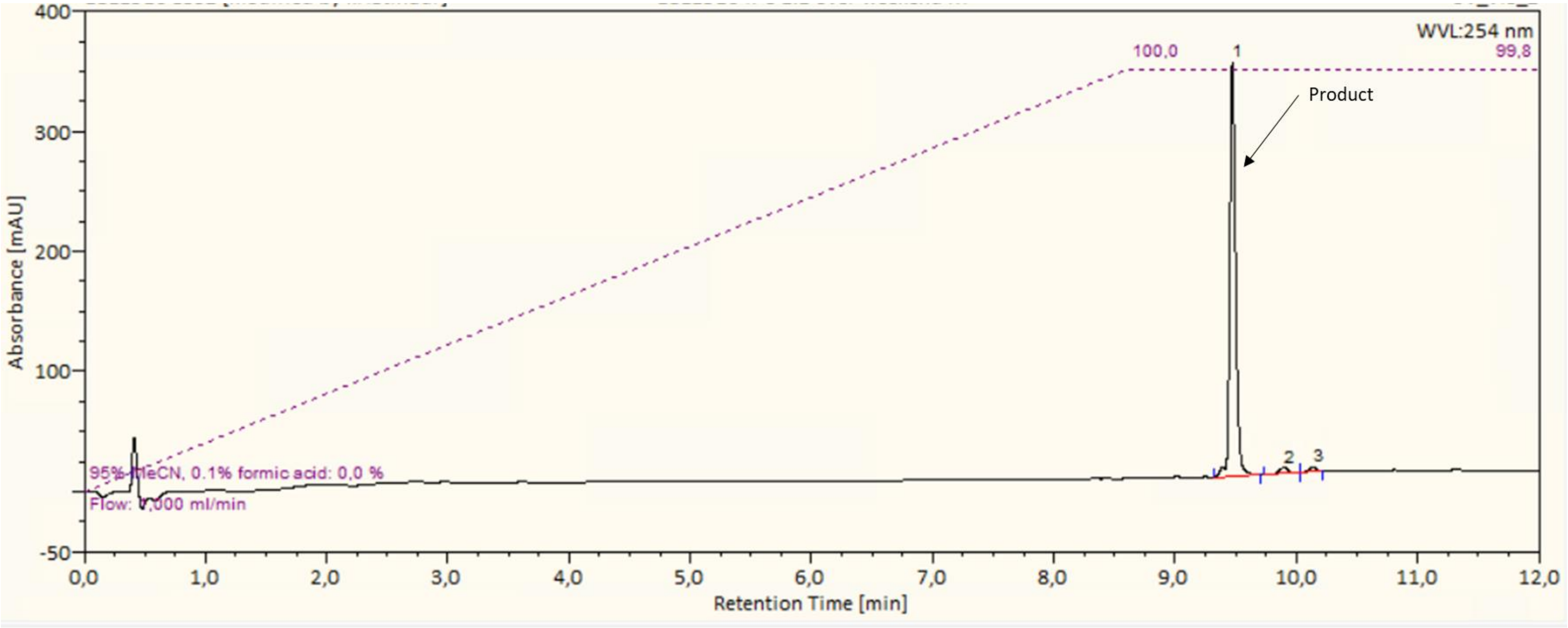

Figure S2. RP-HPLC profile of PTC screen reaction for 2′-OH alkylation of compound **3** *using* K_2CO_3 *as a base and heptane as a solvent on C18 column (Waters XBridge® Oligonucleotide BEH C18 (4.6 x 50 mm)) with a linear gradient from 0-100% of buffer B in buffer A over 12 min at 40 °C. Buffer A: 0.1 M triethylammonium acetate (aq.), buffer B: MeCN+10% (v/v) buffer A.*

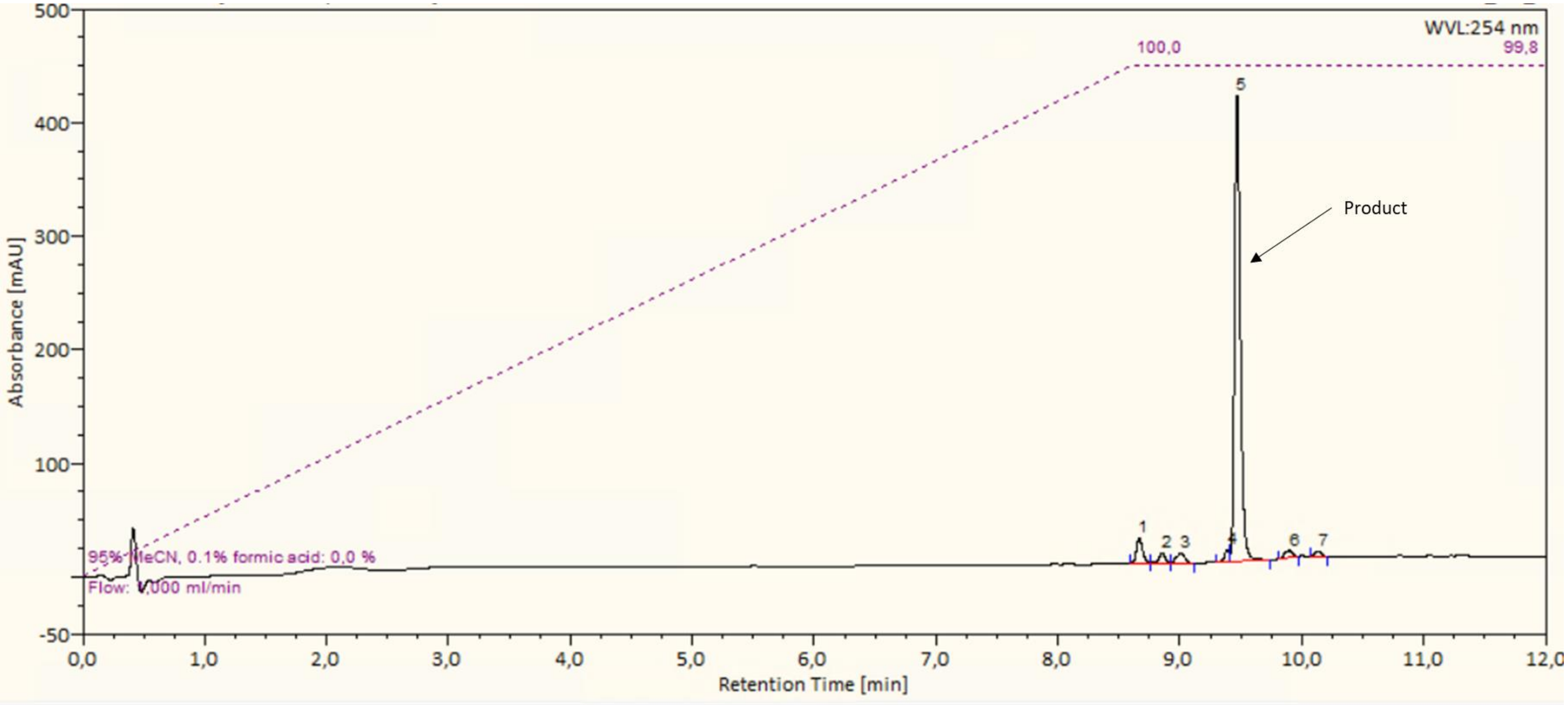

Figure S3. RP-HPLC profile of PTC screen reaction for 2′-OH alkylation of compound **3** *using K_2CO_3 as a base and MeCN as a solvent on C18 column (Waters XBridge® Oligonucleotide BEH C18 (4.6 x 50 mm)) with a linear gradient from 0-100% of buffer B in buffer A over 12 min at 40 °C. Buffer A: 0.1 M triethylammonium acetate (aq.), buffer B: MeCN+10% (v/v) buffer A.*

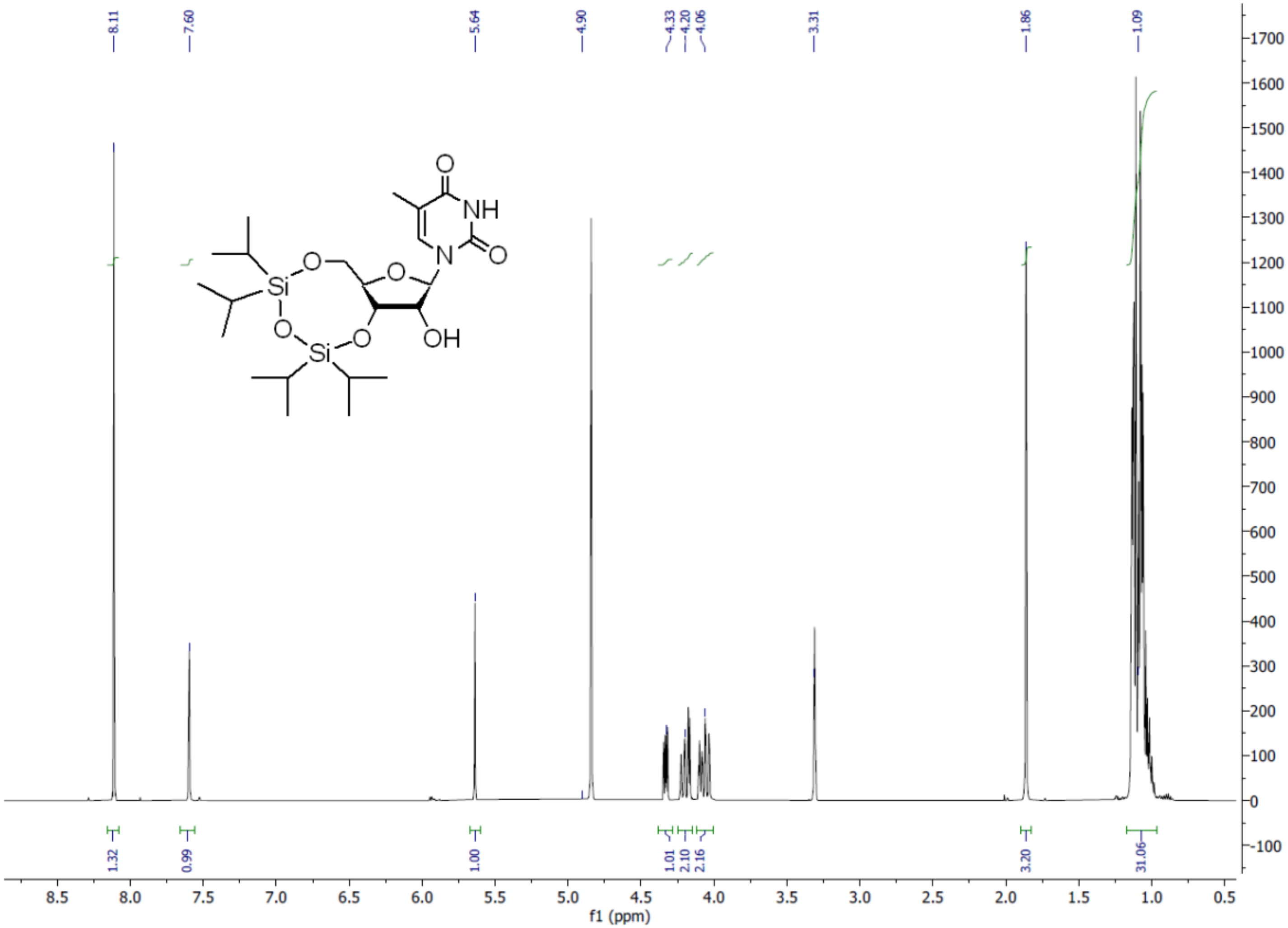

Figure S4. *^{1}H NMR assay of compound* ***1*** *(500 MHz, CD_3OD). 1,2,4,5-Tetrachloro-3-nitrobenzene (δ=8.11) was used as an internal standard.*

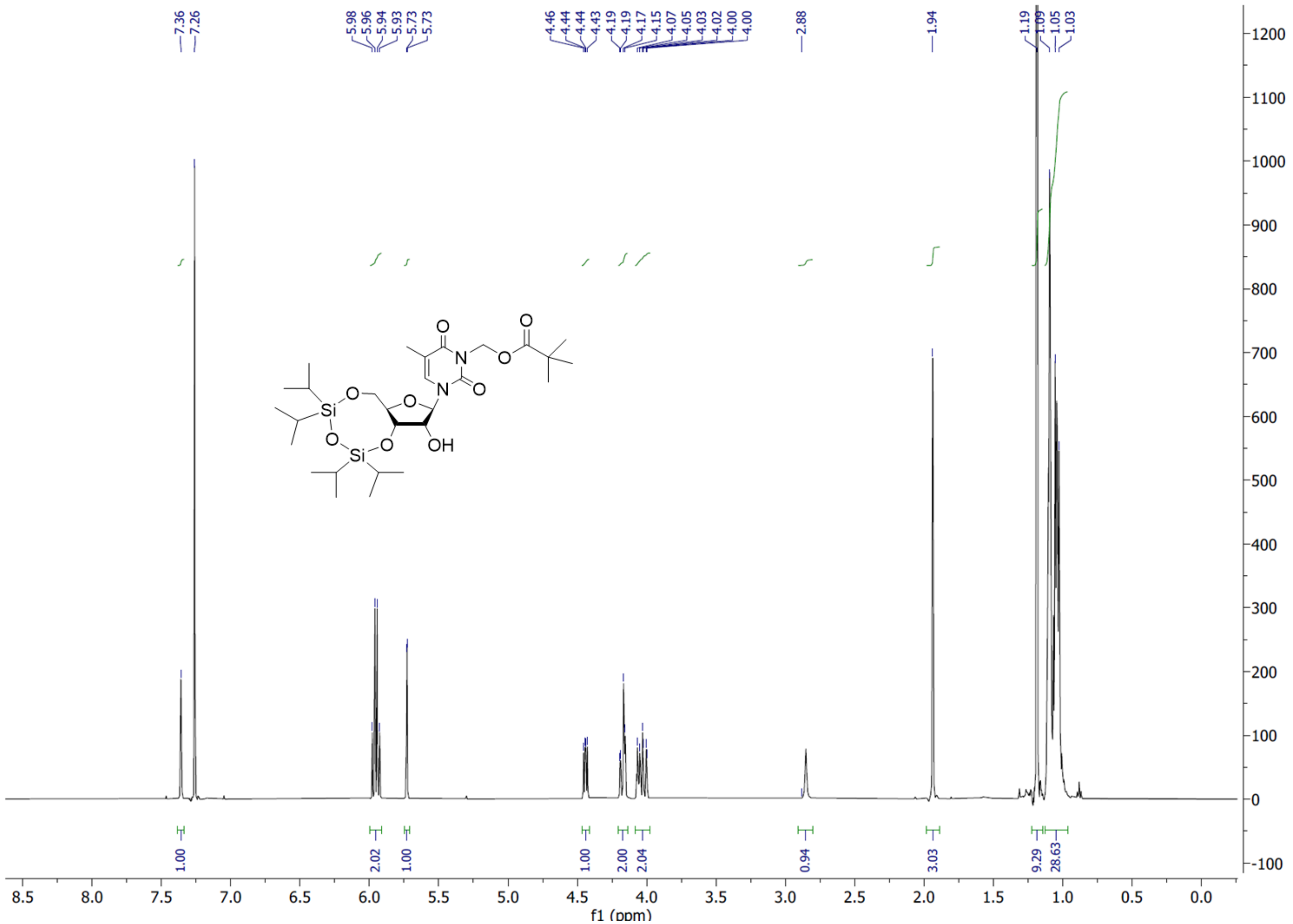

Figure S5. ¹H NMR spectrum of compound **2** *(500 MHz, CDCl₃).*

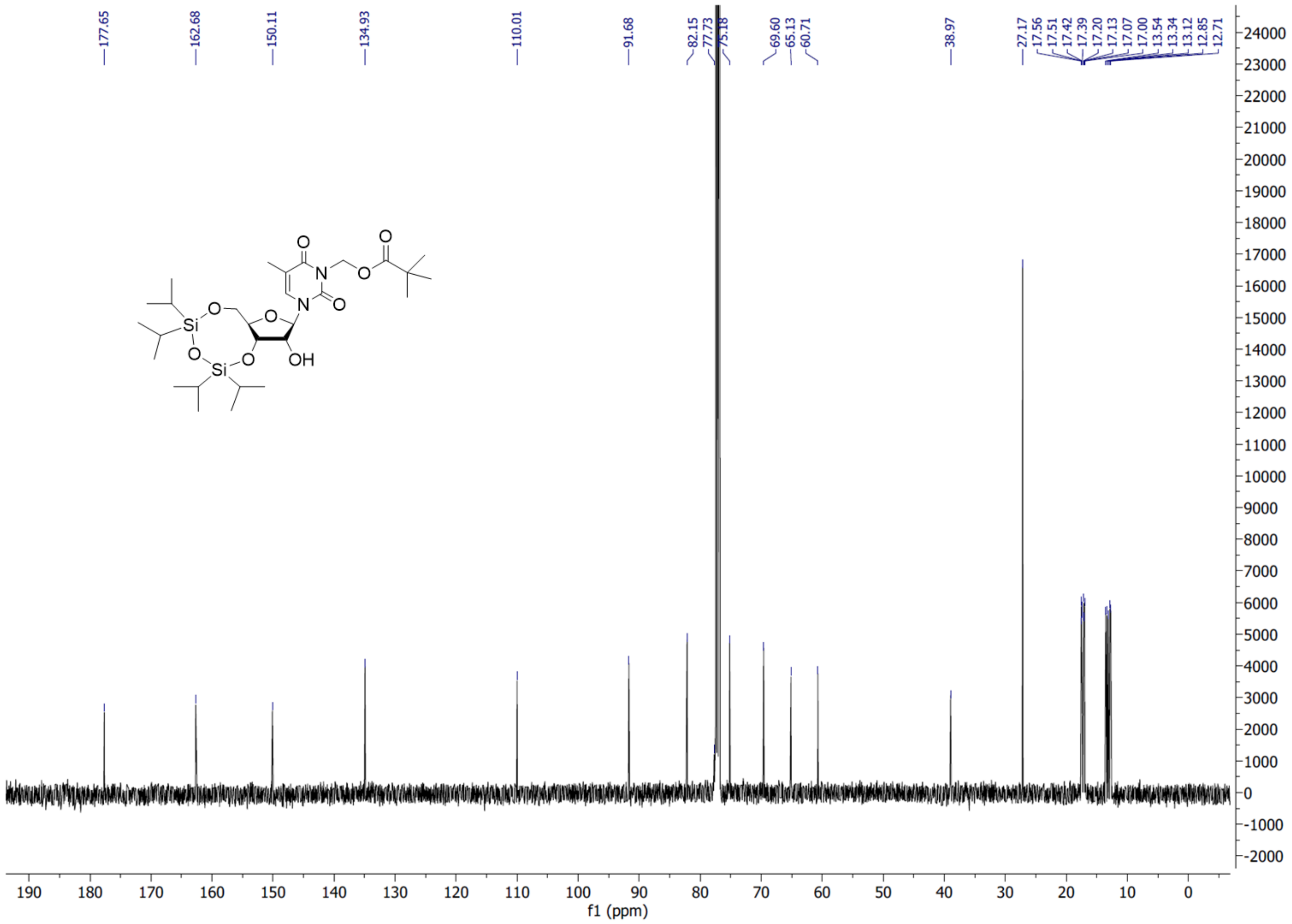

Figure S6. ^{13}C *NMR spectrum of compound* ***2*** *(125.76 MHz,* $CDCl_3$*).*

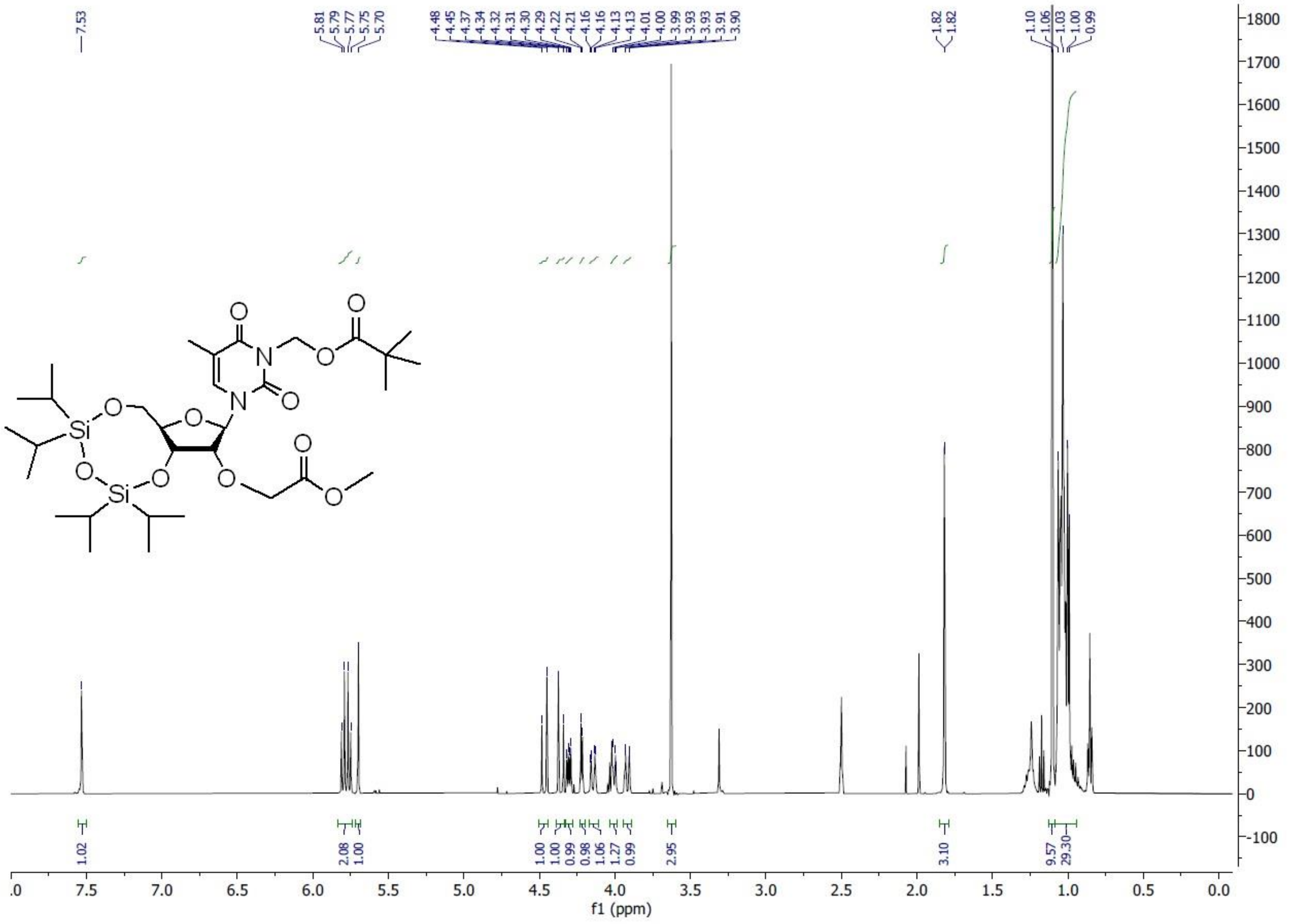

Figure S7. ¹H NMR spectrum of compound **3** (500 MHz, DMSO-d_6).

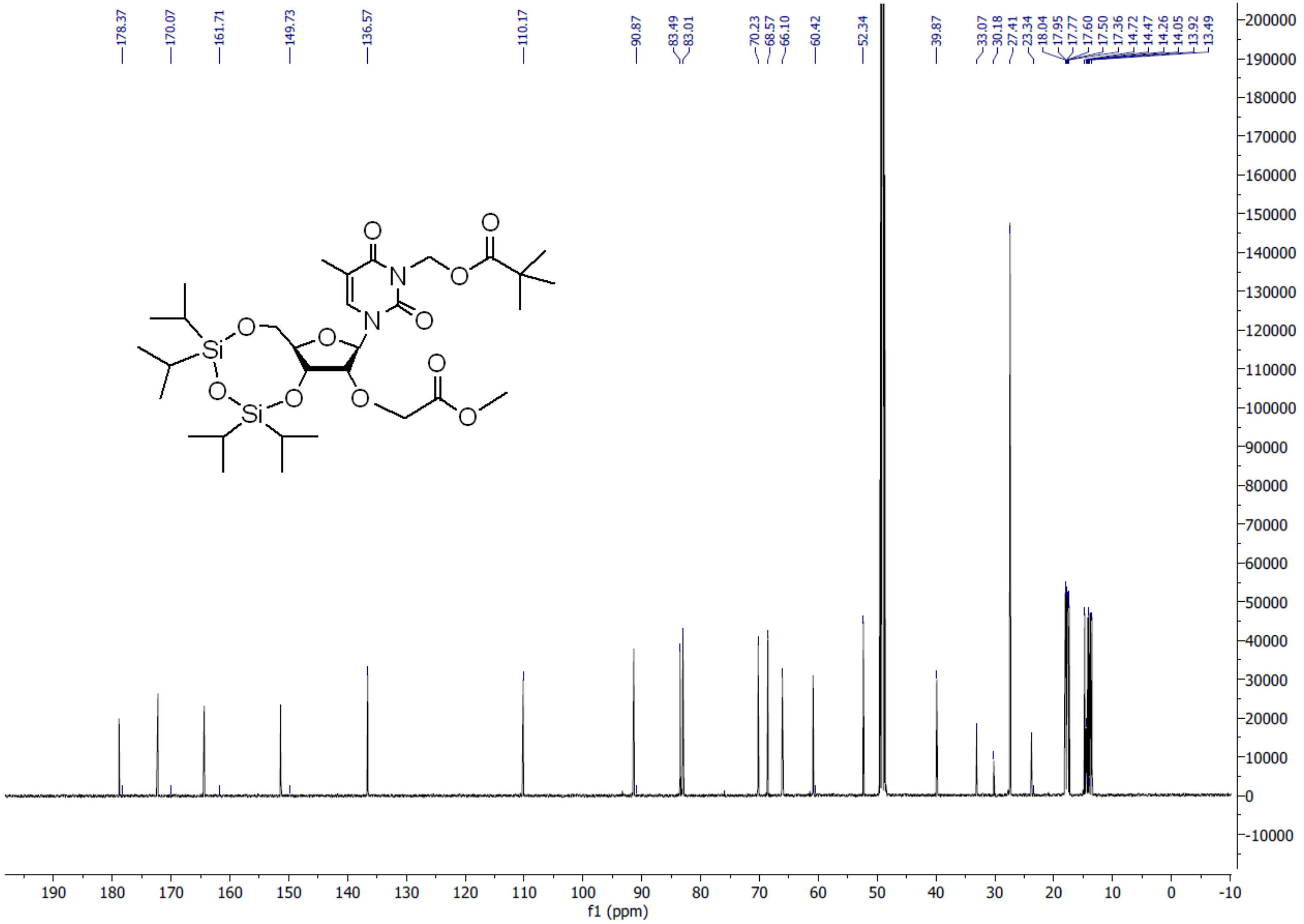

Figure S8. *^{13}C NMR spectrum of compound* ***3*** *(125.76 MHz, CD$_3$OD).*

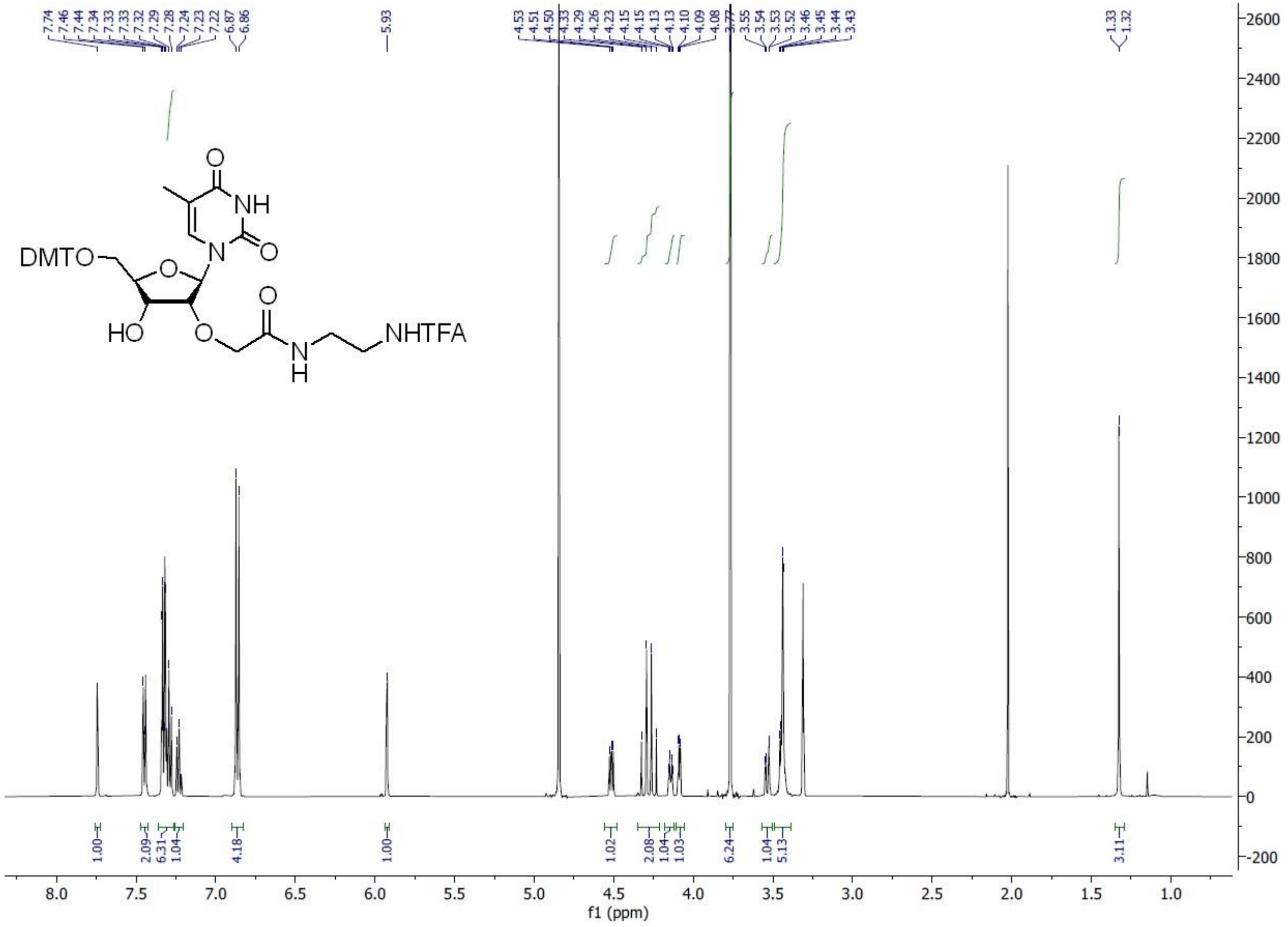

*Figure S9. ¹H NMR spectrum of compound **6** (500 MHz, CD_3OD).*

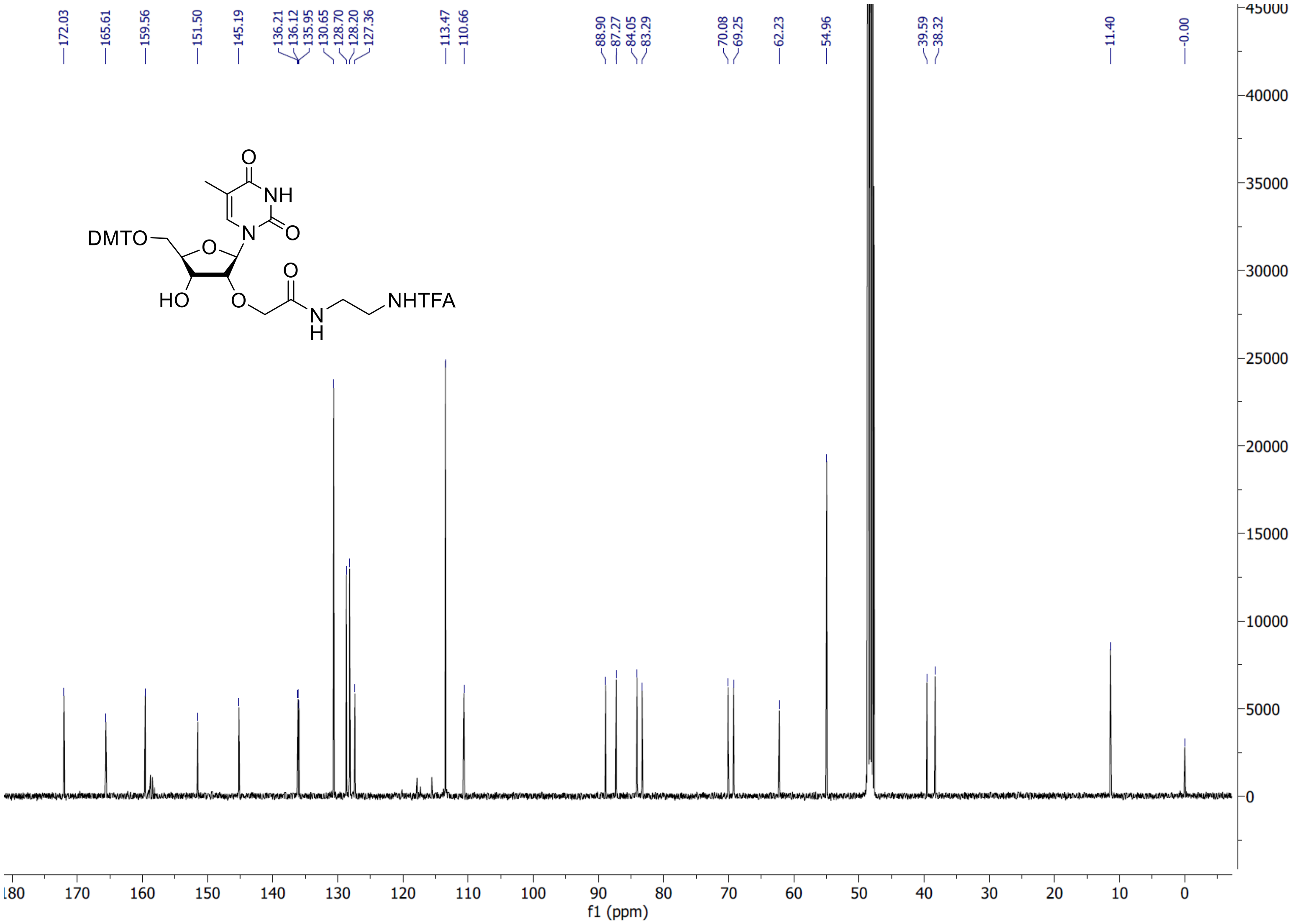

Figure S10. [13]*C NMR spectrum of compound* ***6*** *(125.76 MHz, CD*$_3$*OD).*

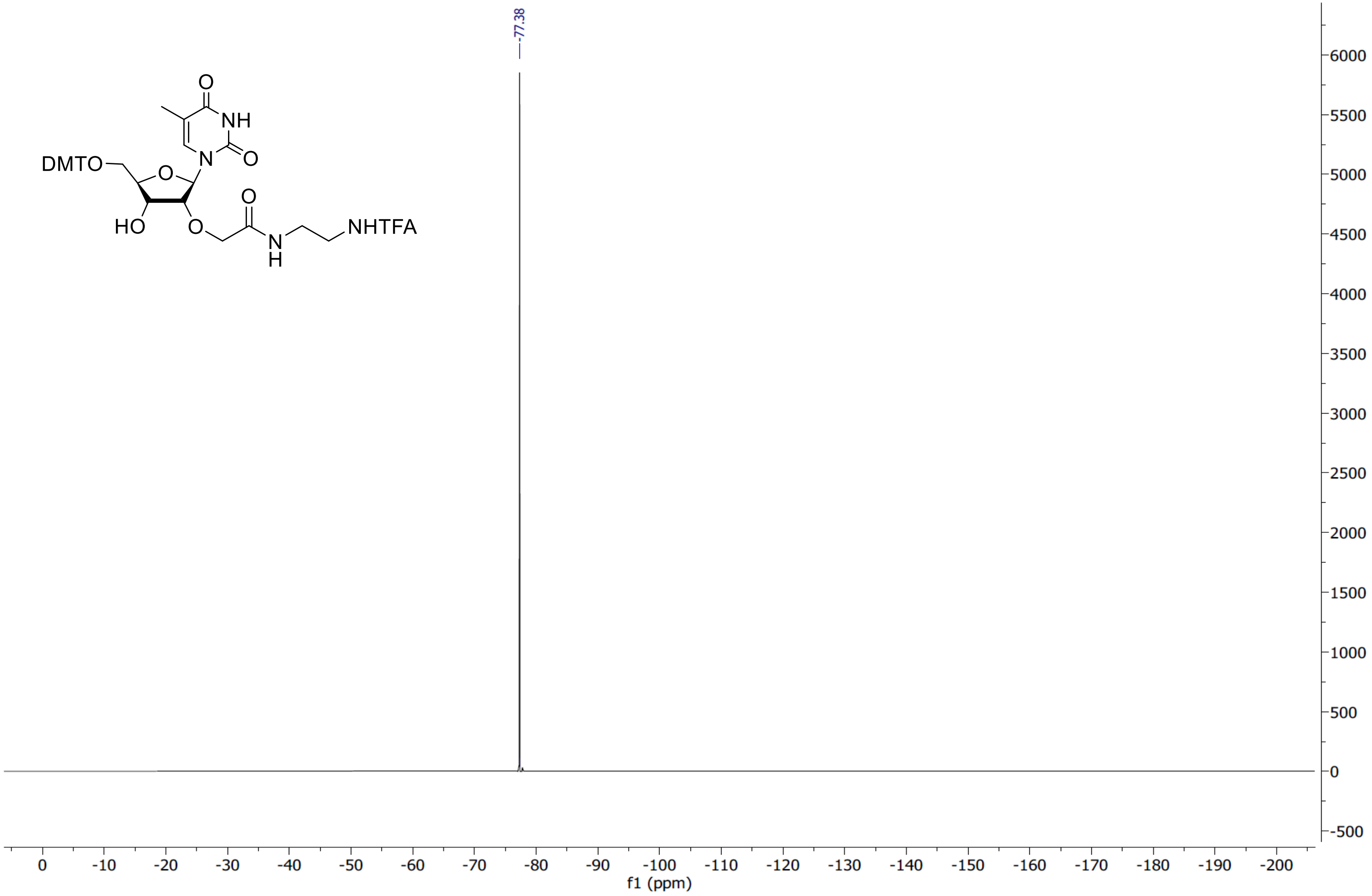

Figure S11. *^{19}F NMR spectrum of compound* ***6*** *(470.56 MHz, CD_3OD).*

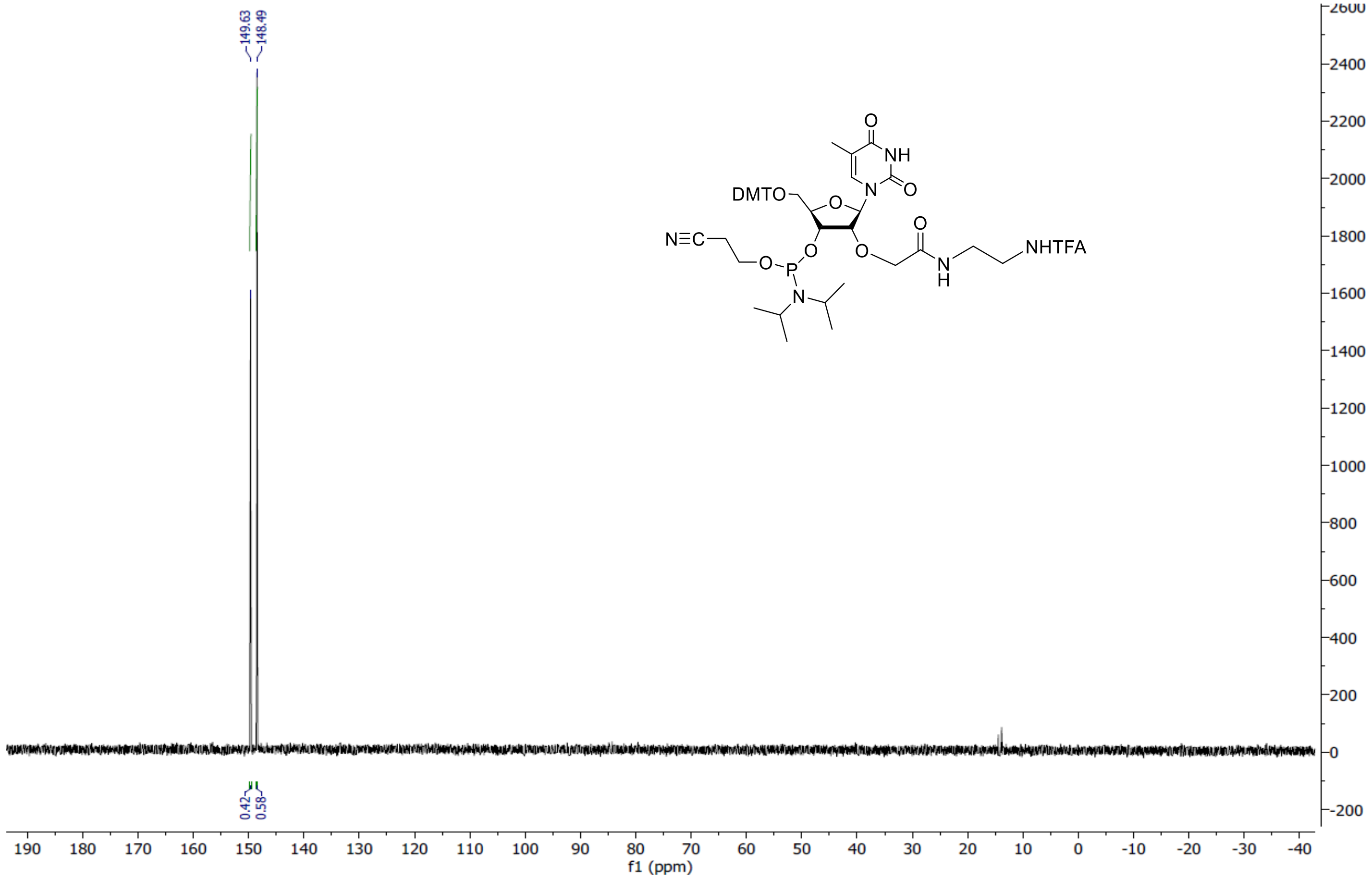

Figure S12. ^{31}*P NMR spectrum of compound* ***7*** *(202.47 MHz, CD_3CN).*

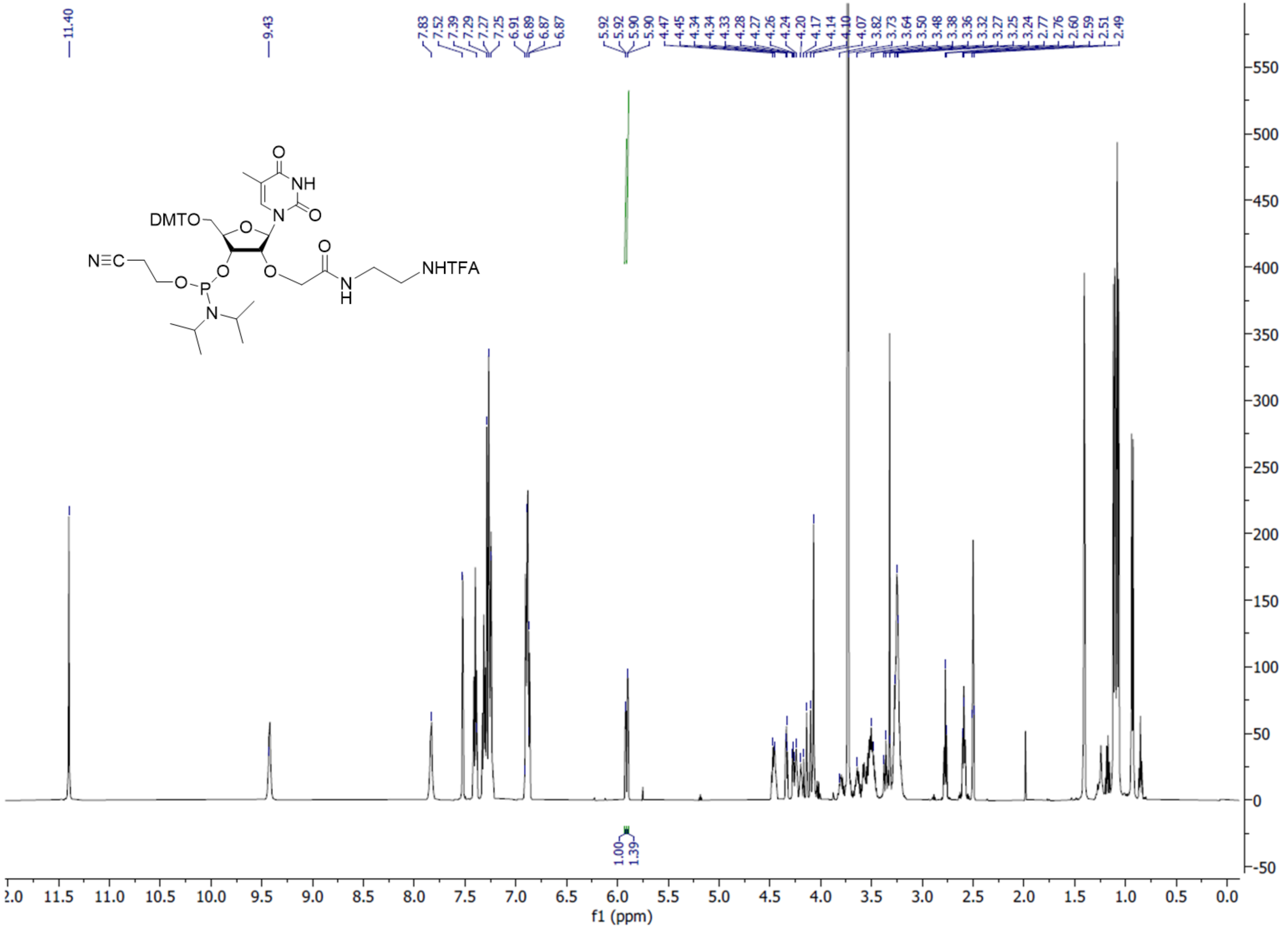

*Figure S13 .^{1}H NMR spectrum of compound **7** (500 MHz, DMSO-d6).*

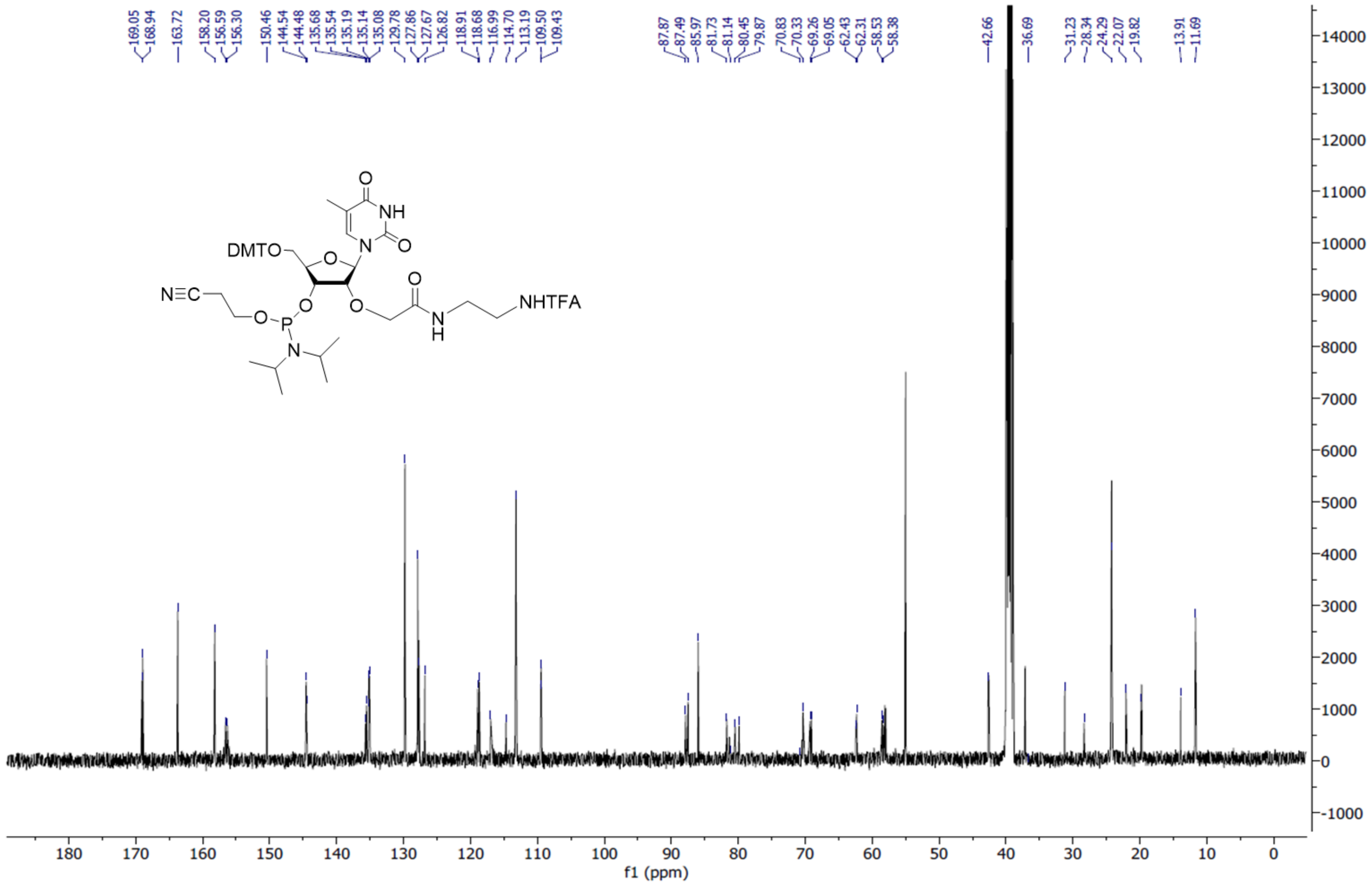

Figure S14. *^{13}C NMR spectrum of compound* ***7*** *(125.76 MHz, DMSO-d₆).*

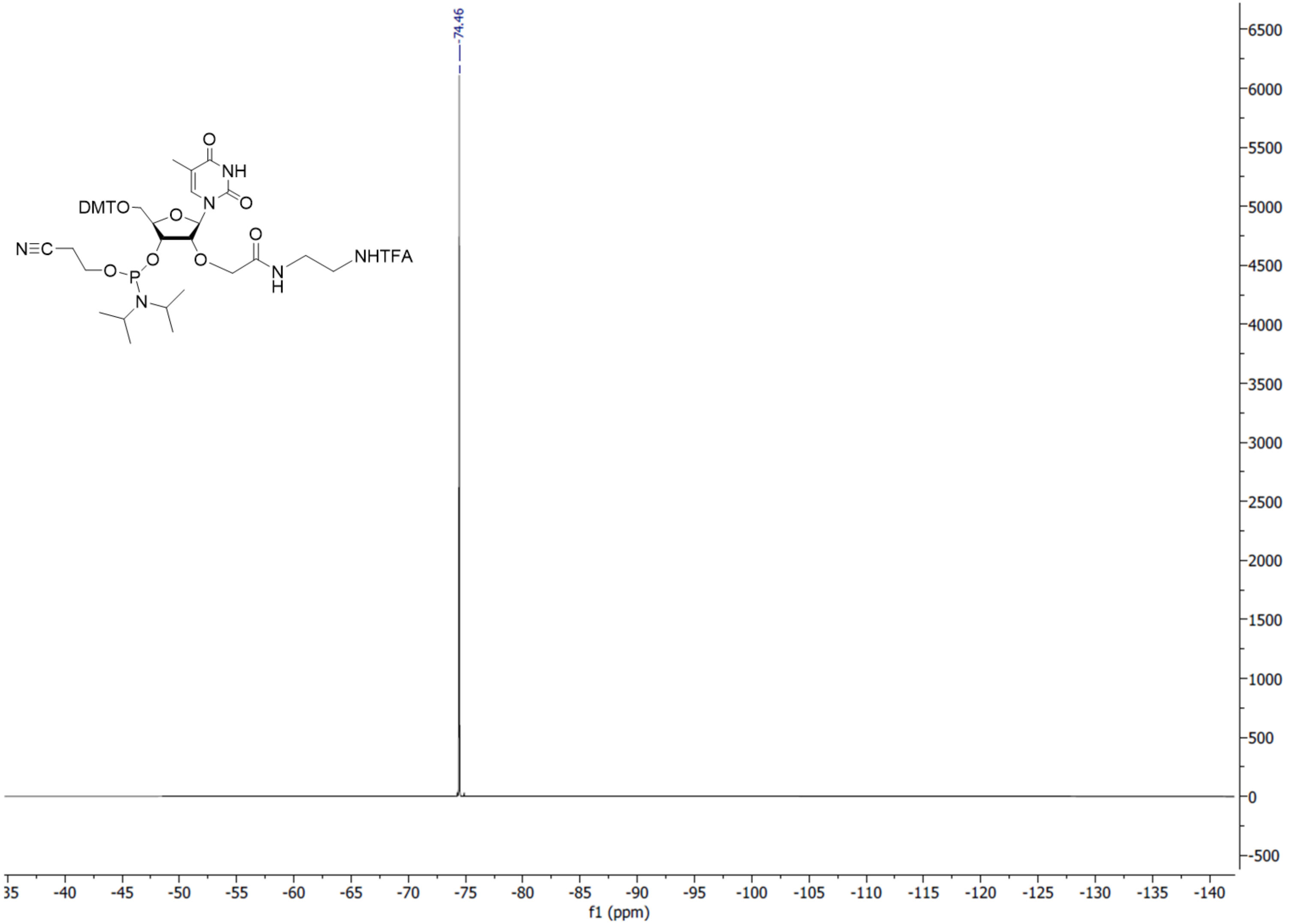

*Figure S15. ^{19}F NMR spectrum of compound **7** (470.56 MHz, DMSO-d6).*

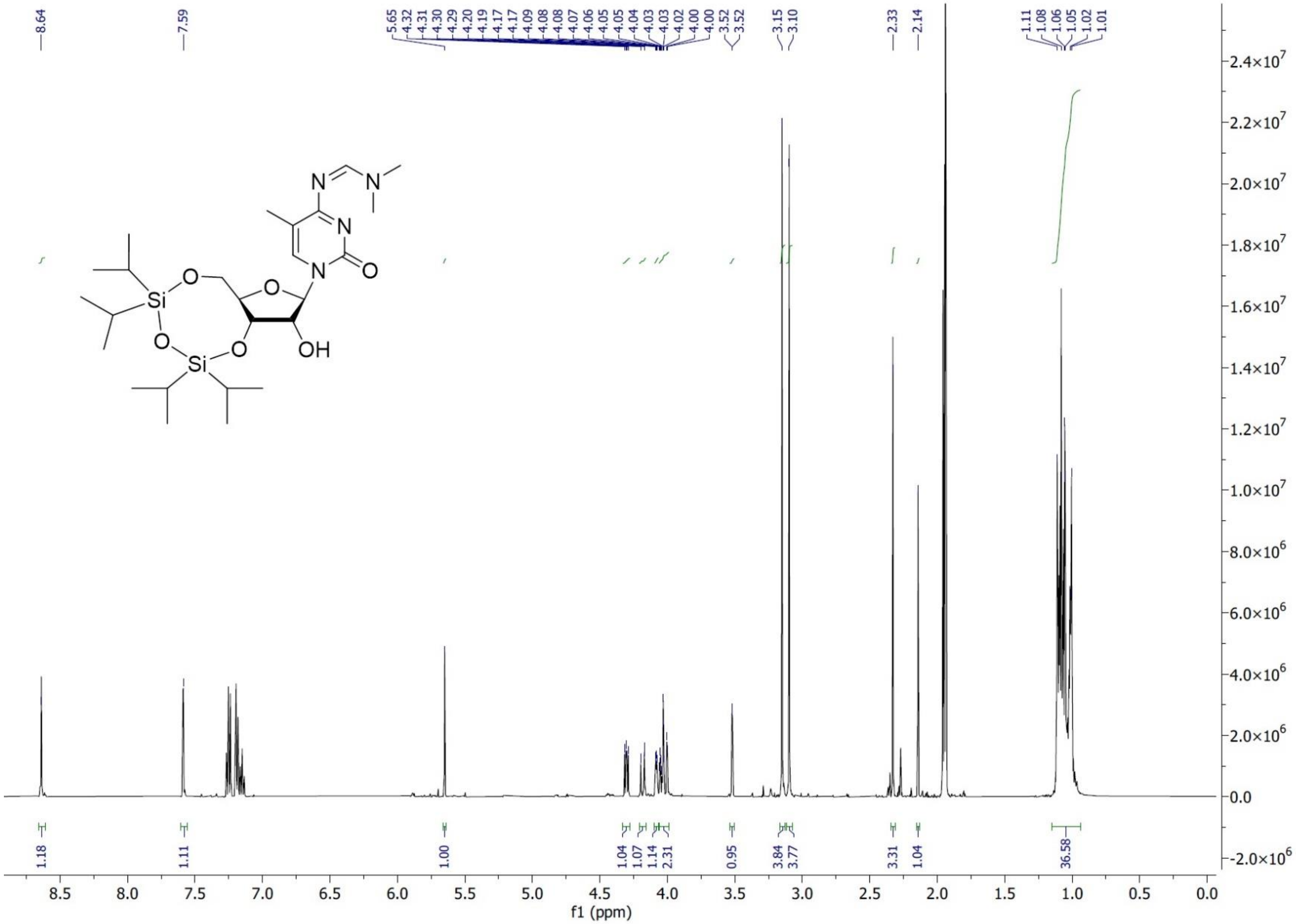

Figure S16. *¹H NMR spectrum of crude compound* ***9*** *(500 MHz, CD_3CN).*

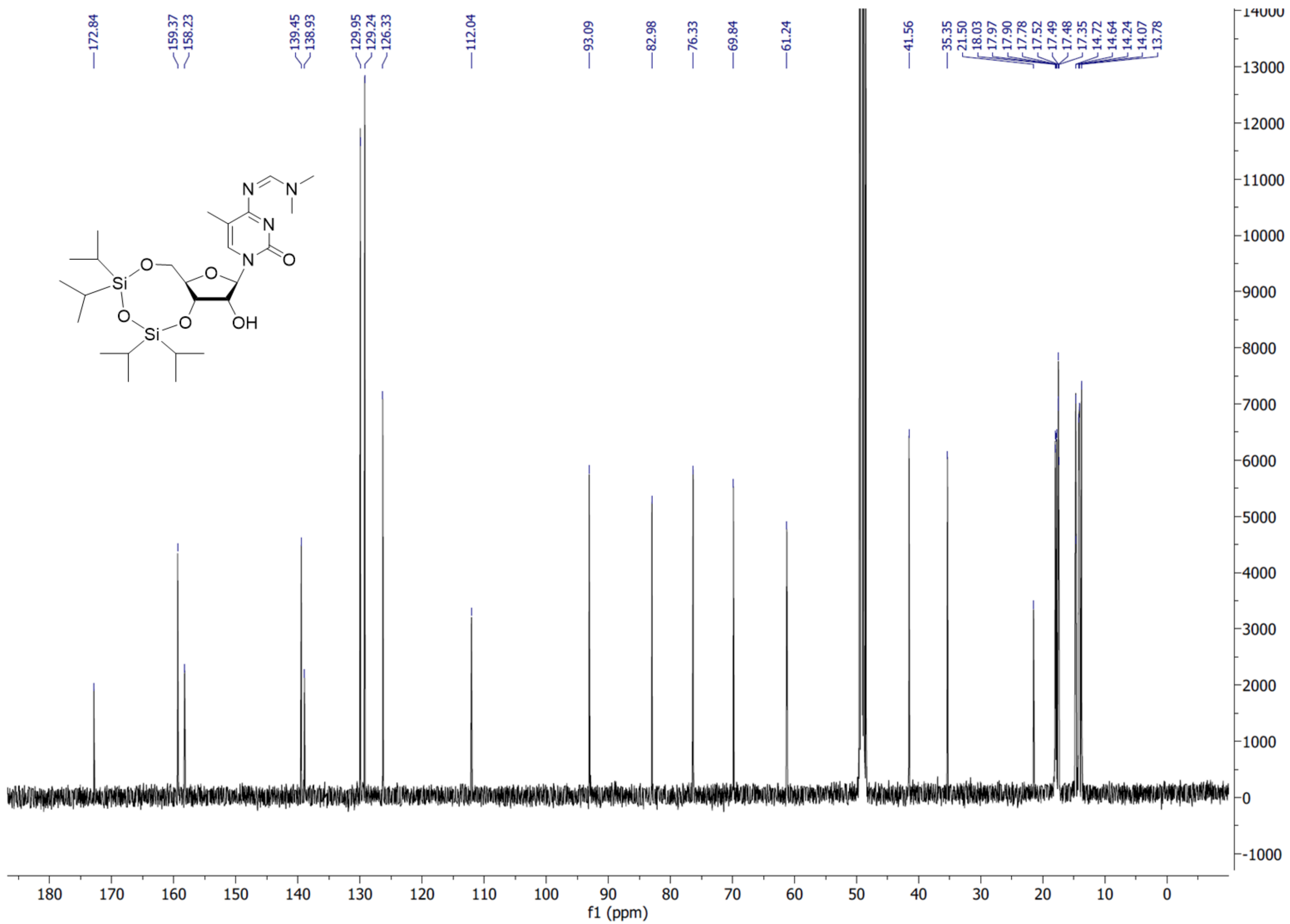

Figure S17. ^{13}C NMR spectrum of crude compound ***9*** *(125.76 MHz, CD_3OD).*

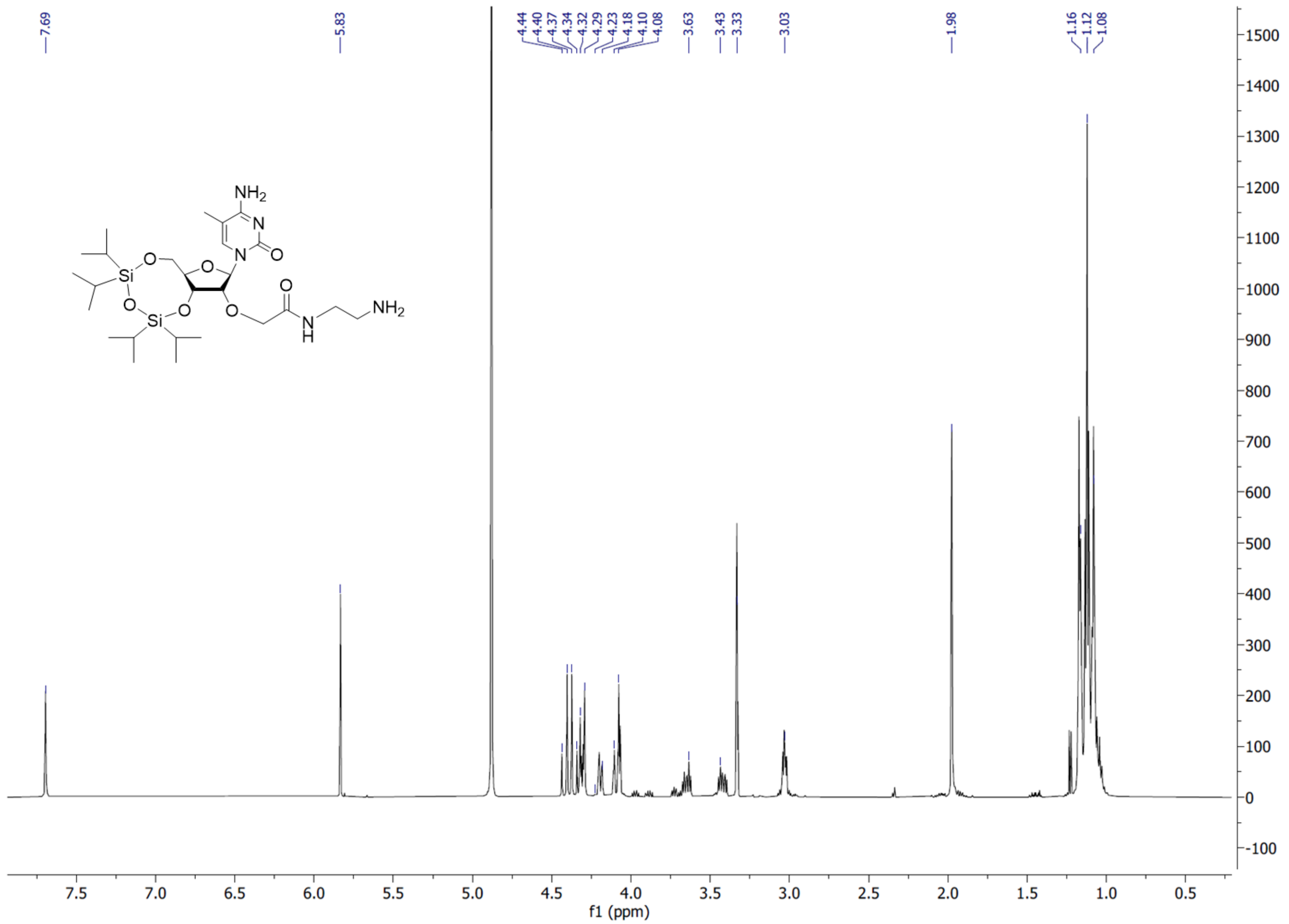

Figure S18. ¹H NMR spectrum of crude compound ***11*** *(500 MHz, CD_3OD).*

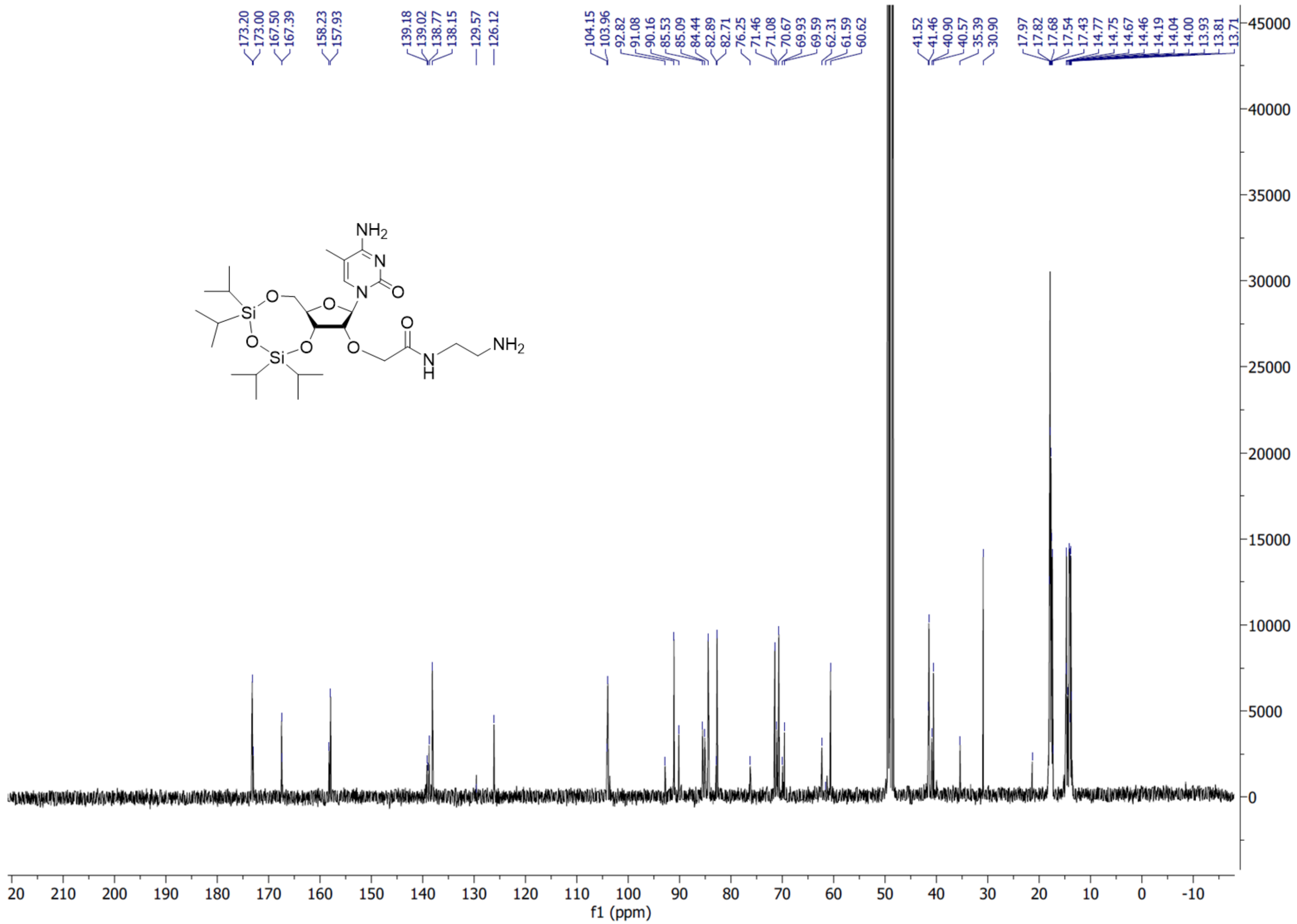

*Figure S19. ^{13}C NMR spectrum of crude compound **11** (125.76 MHz, CD_3OD).*

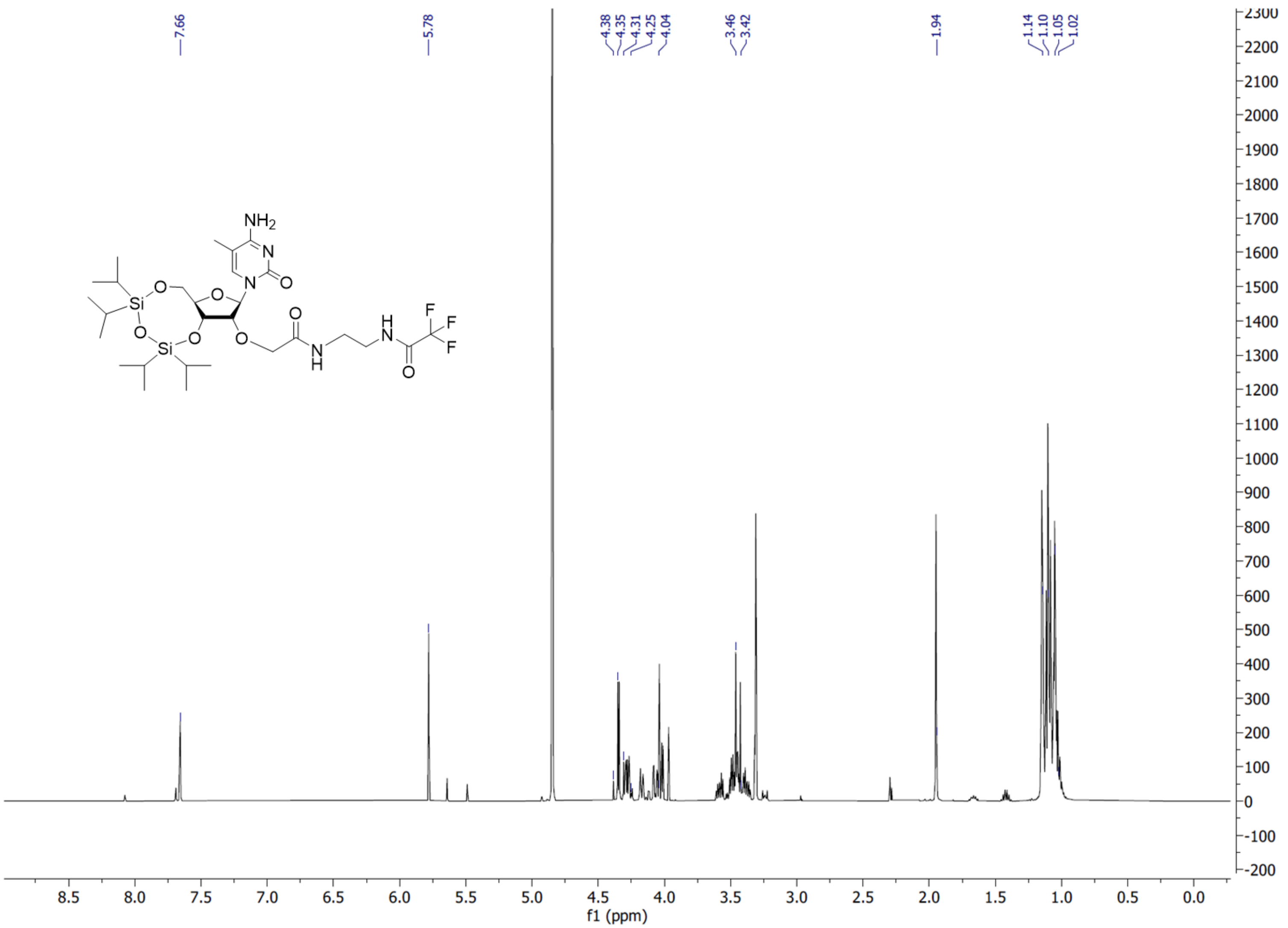

*Figure S20. ^{1}H NMR spectrum of crude compound **12** (500 MHz, CD_3OD).*

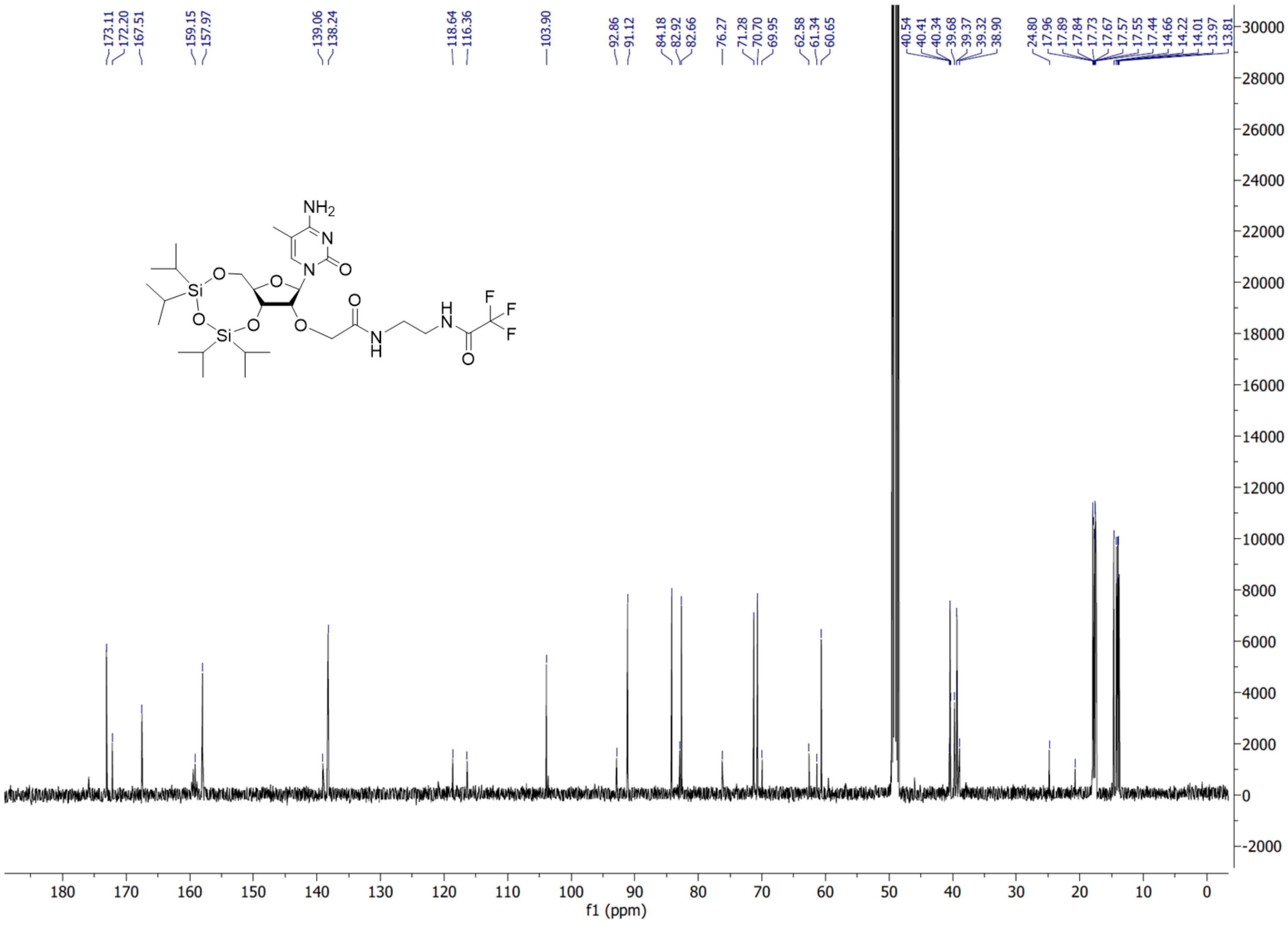

*Figure S21. ^{13}C NMR spectrum of crude compound **12** (125.76 MHz, CD_3OD).*

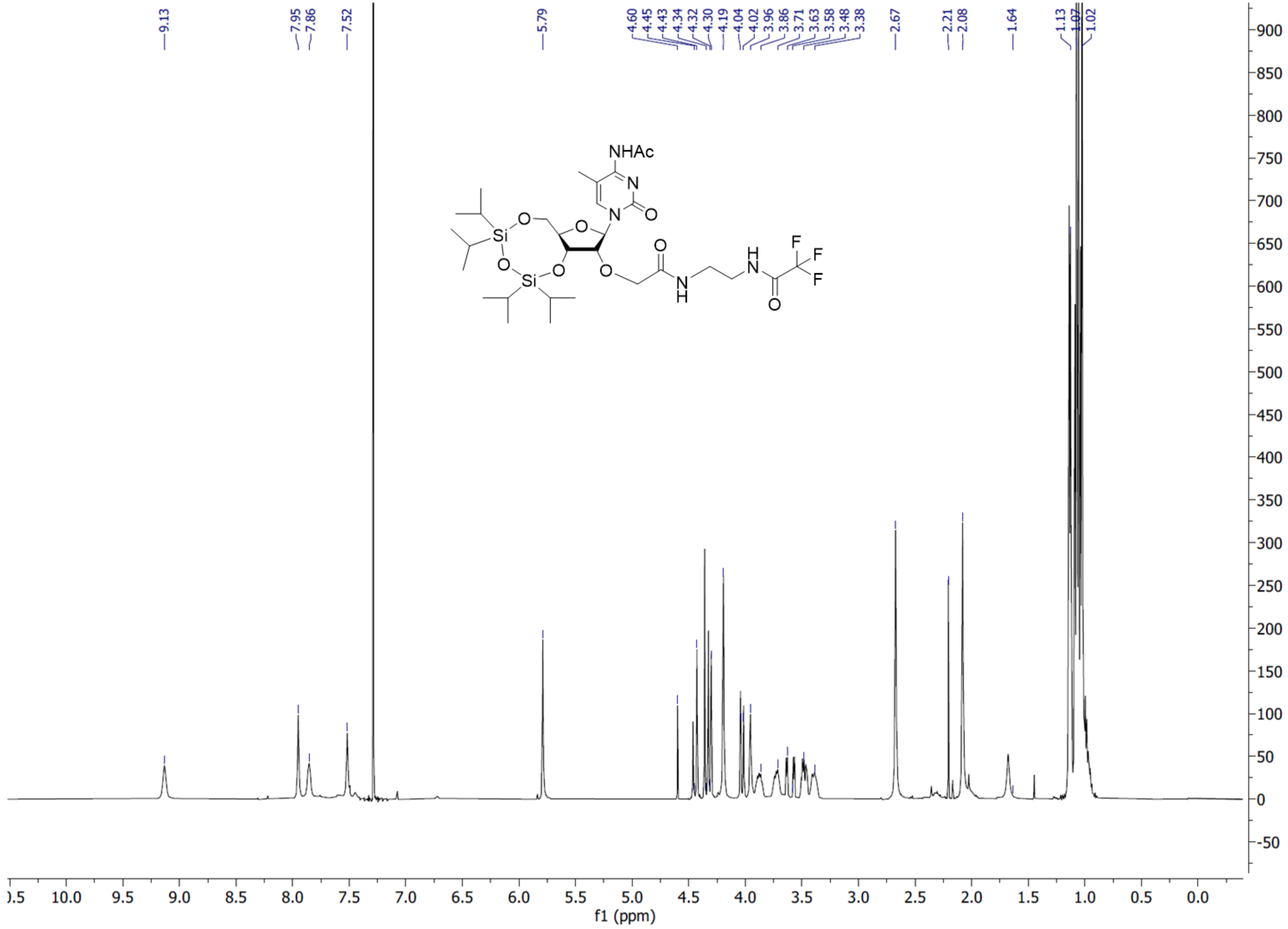

*Figure S22. ¹H NMR spectrum of crude compound **13** (500 MHz, CDCl₃).*

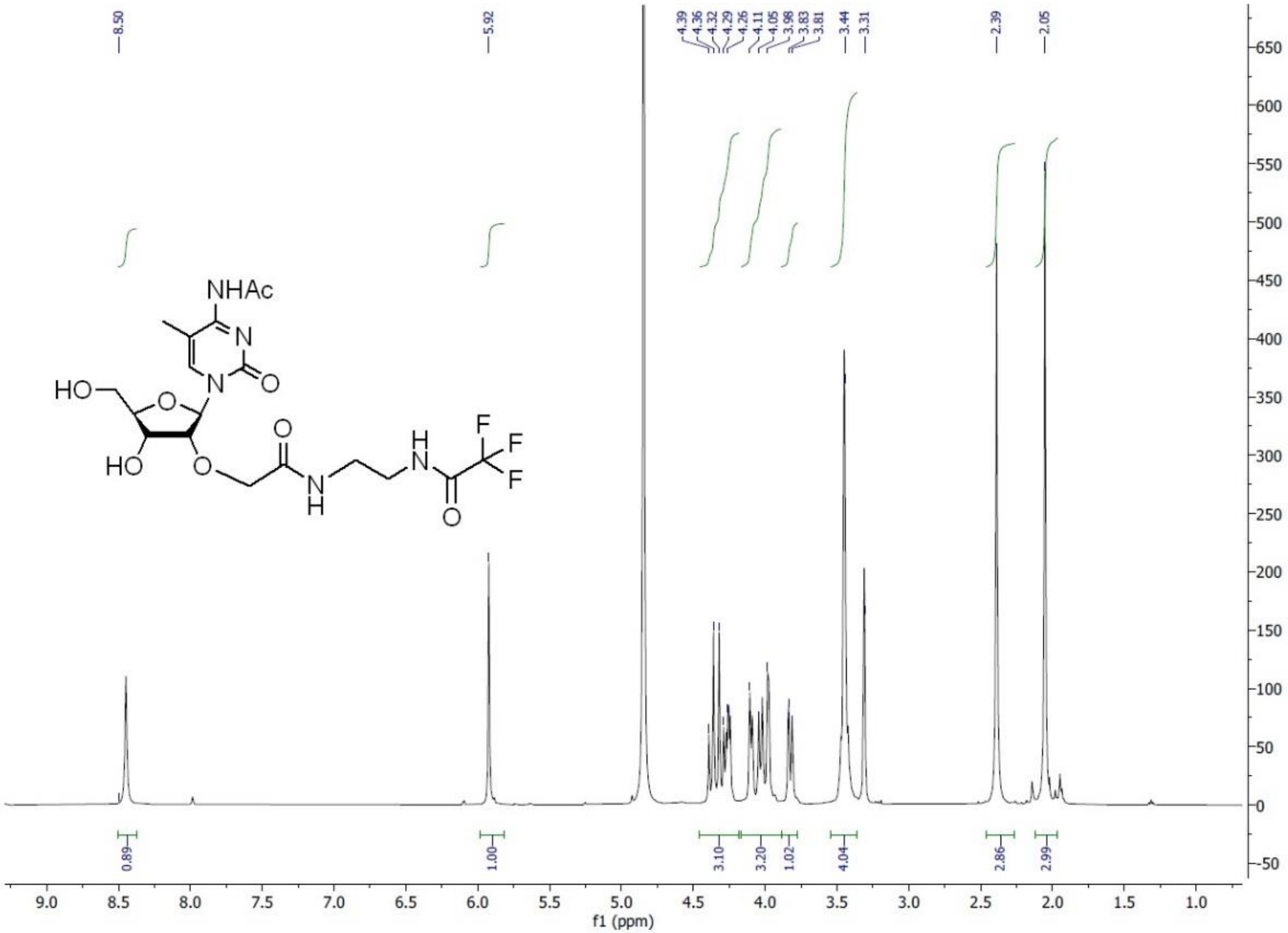

*Figure S23. ¹H NMR spectrum of compound **14** (500 MHz, CD₃OD).*

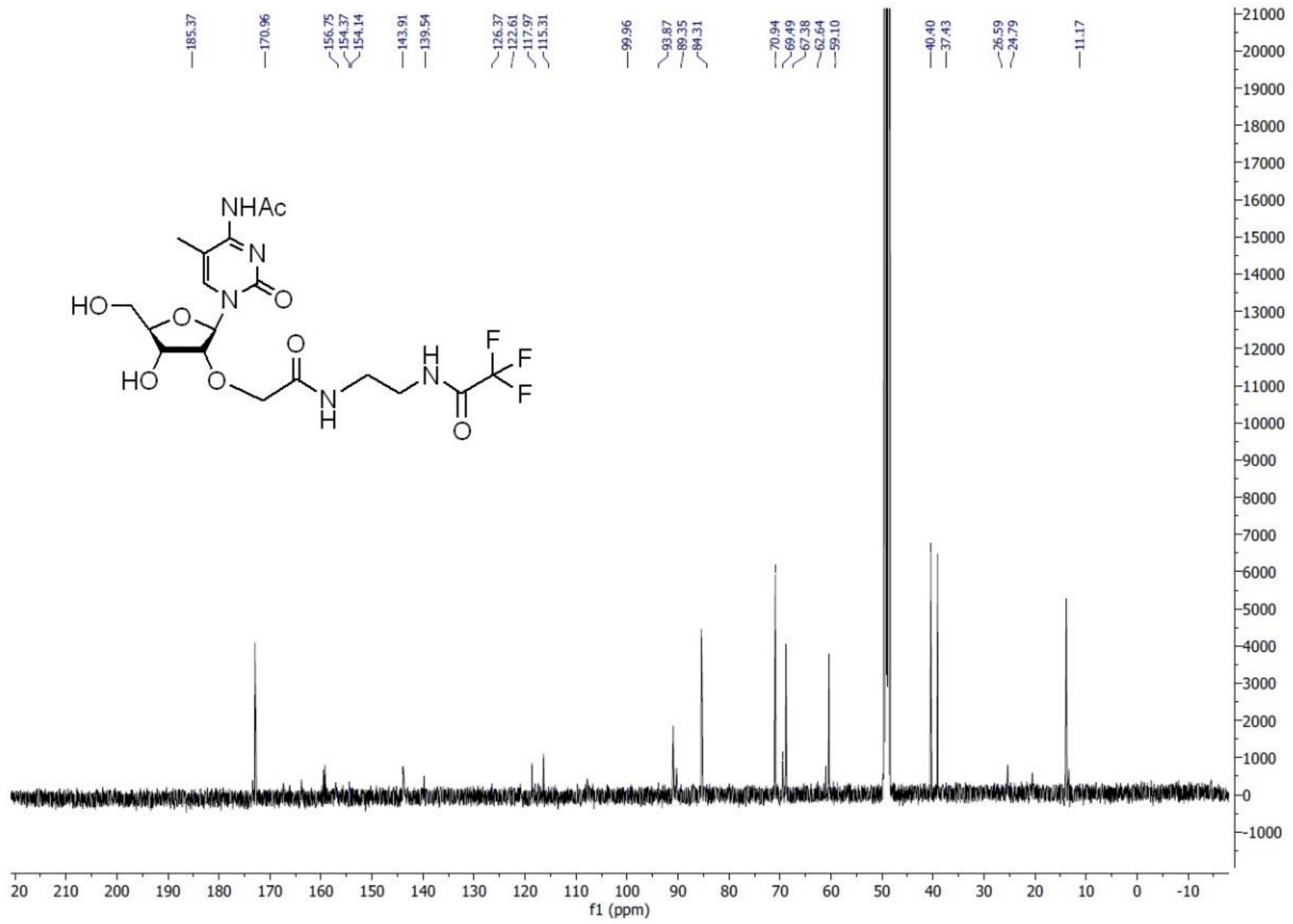

*Figure S24. ^{13}C NMR spectrum of compound **14** (125.76 MHz, CD3OD).*

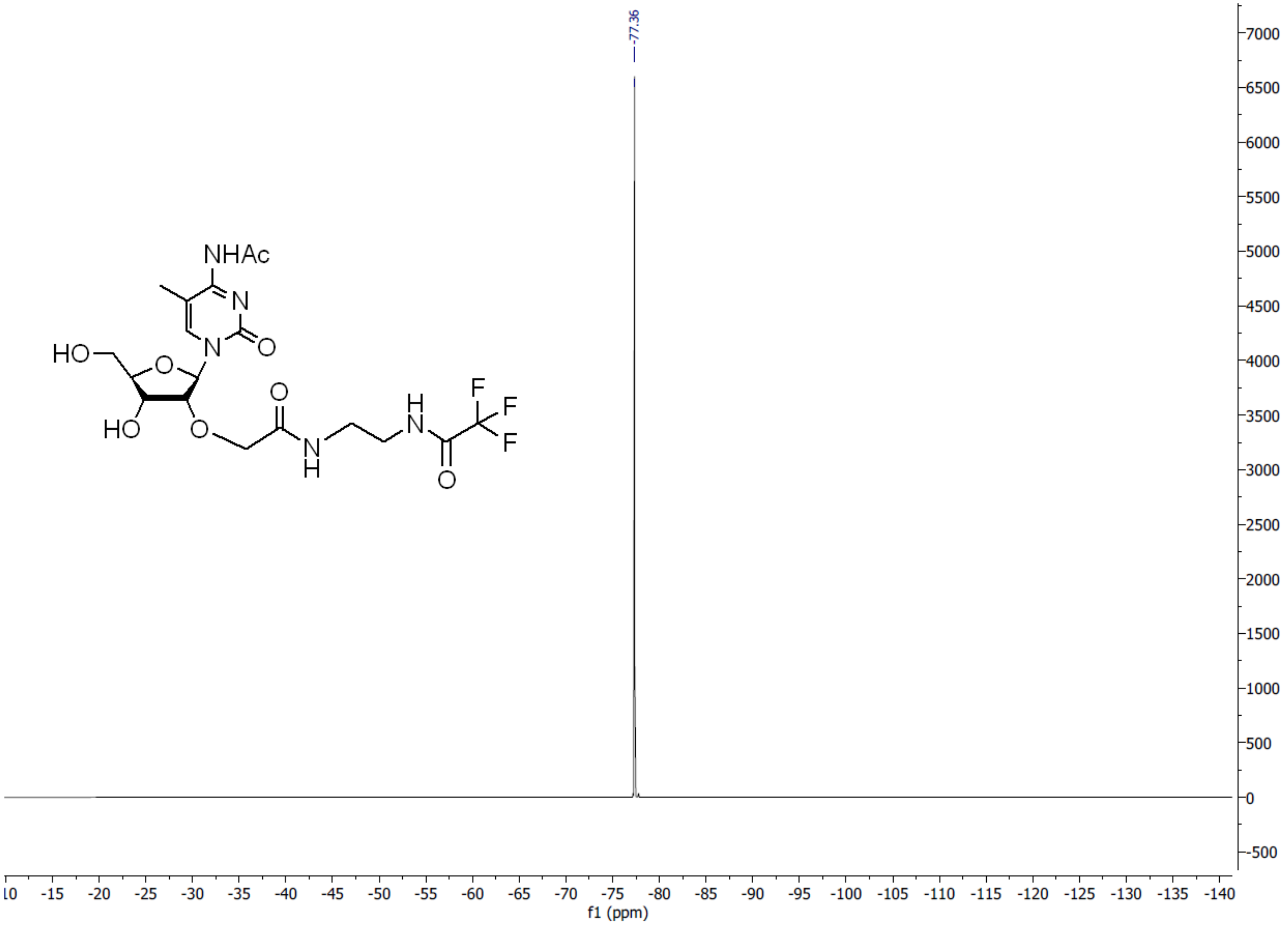

*Figure S25. ^{19}F NMR spectrum of compound **14** (470.56 MHz, CD_3OD).*

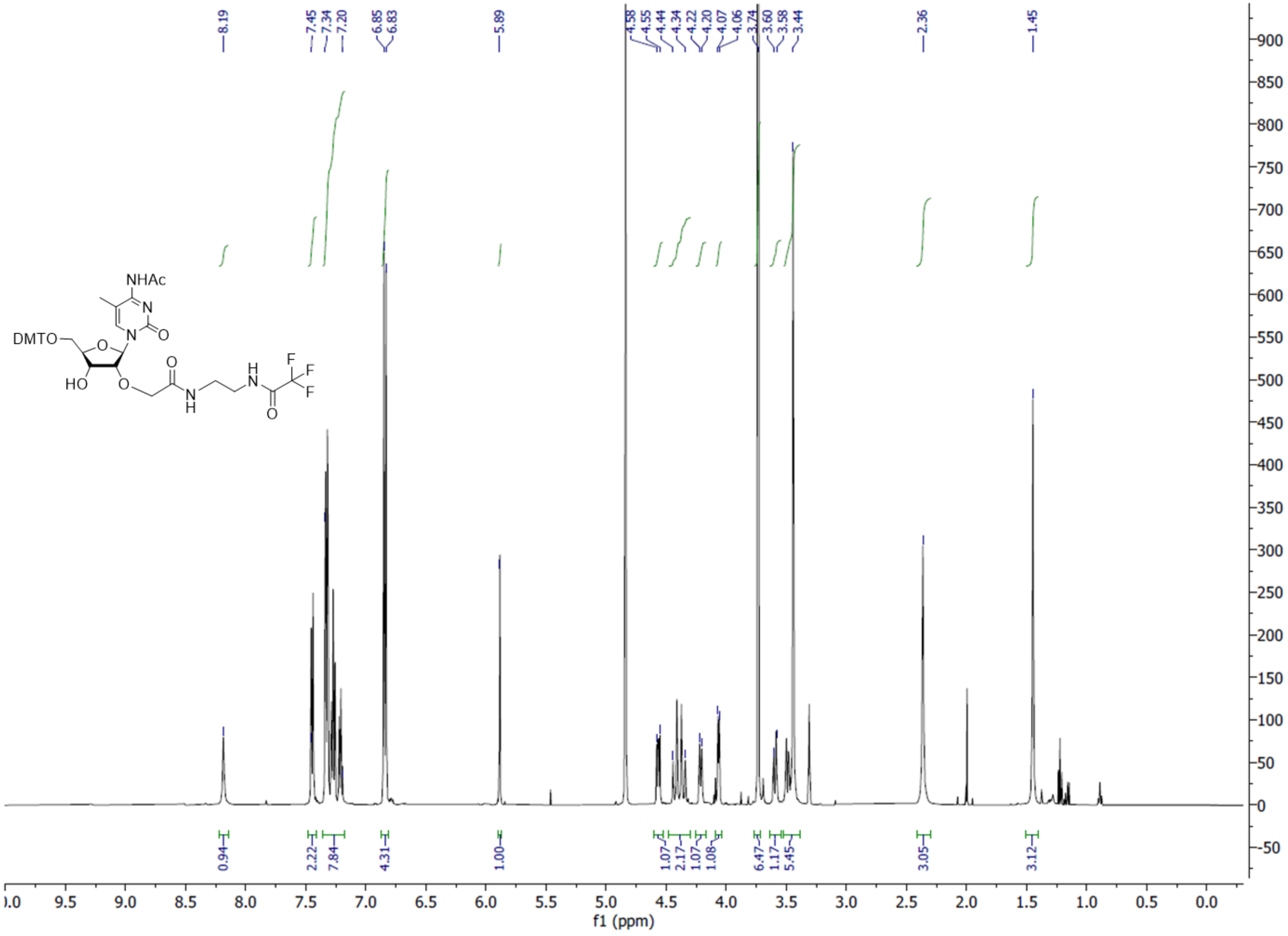

Figure S26. ¹H NMR spectrum of compound ***15*** *(500 MHz, CD_3OD)*

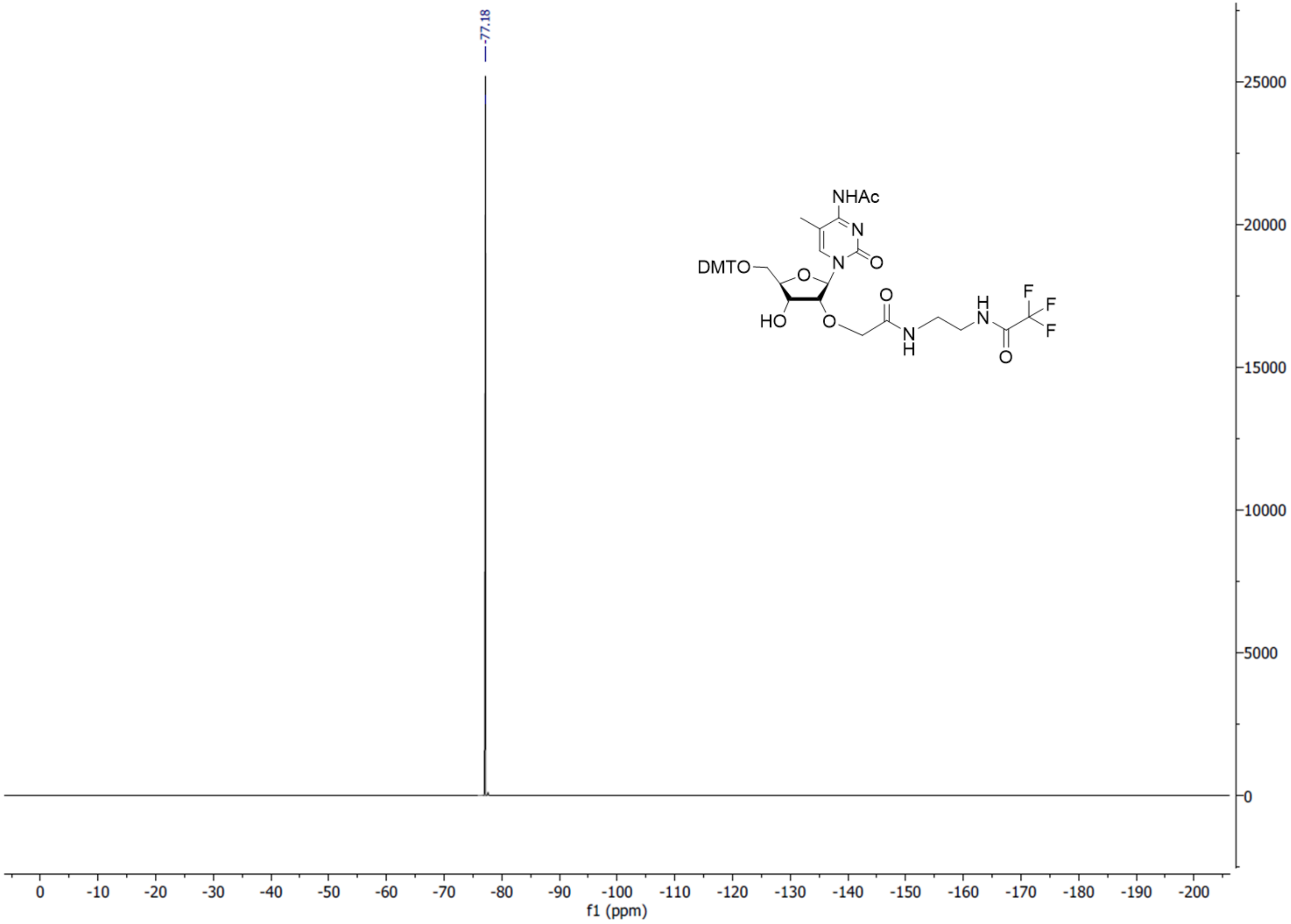

*Figure S27. ^{19}F NMR spectrum of compound **15** (470.56 MHz, CD_3OD).*

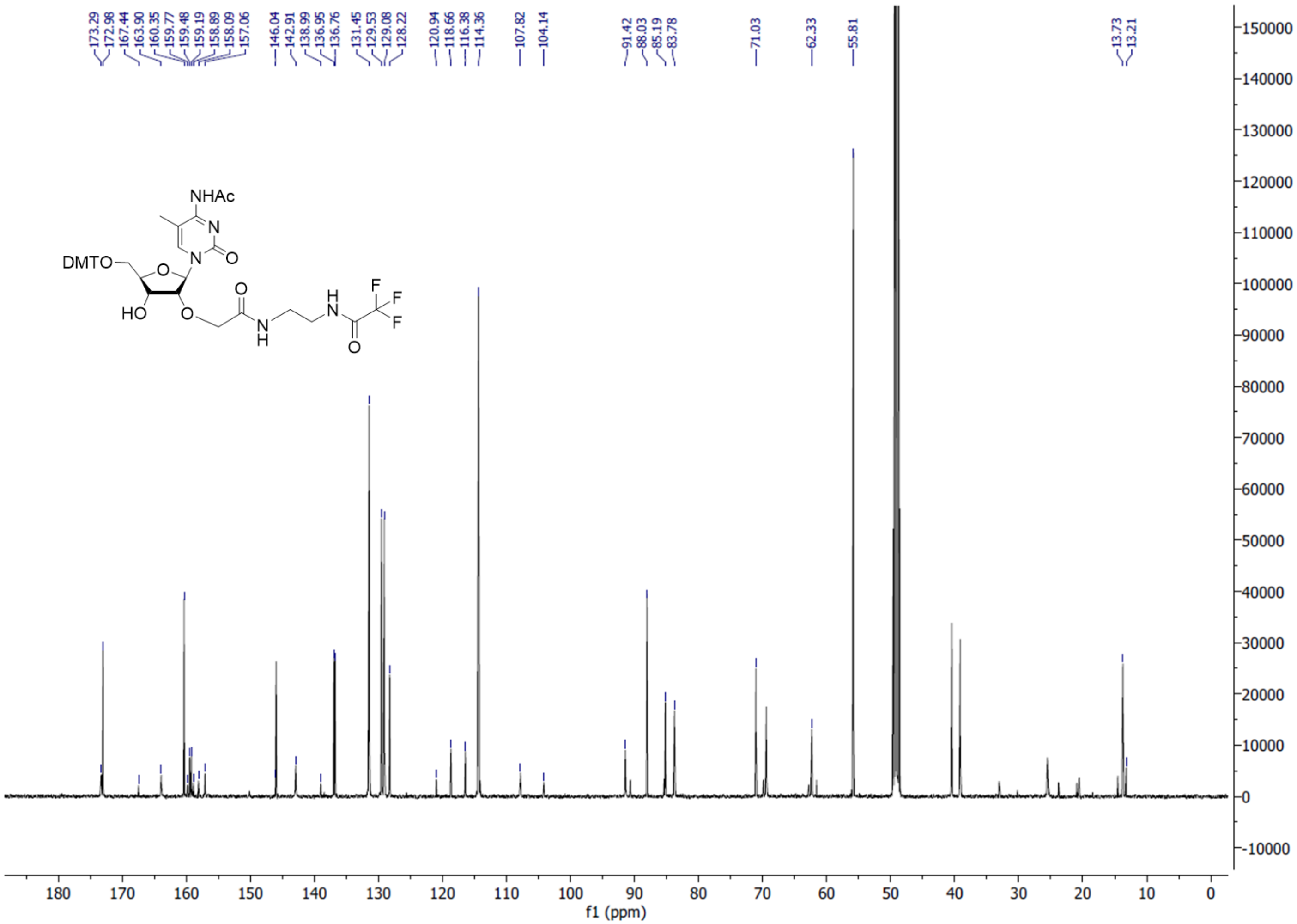

Figure S28. ^{13}C NMR spectrum of compound ***15*** *(125.76 MHz, CD3OD).*

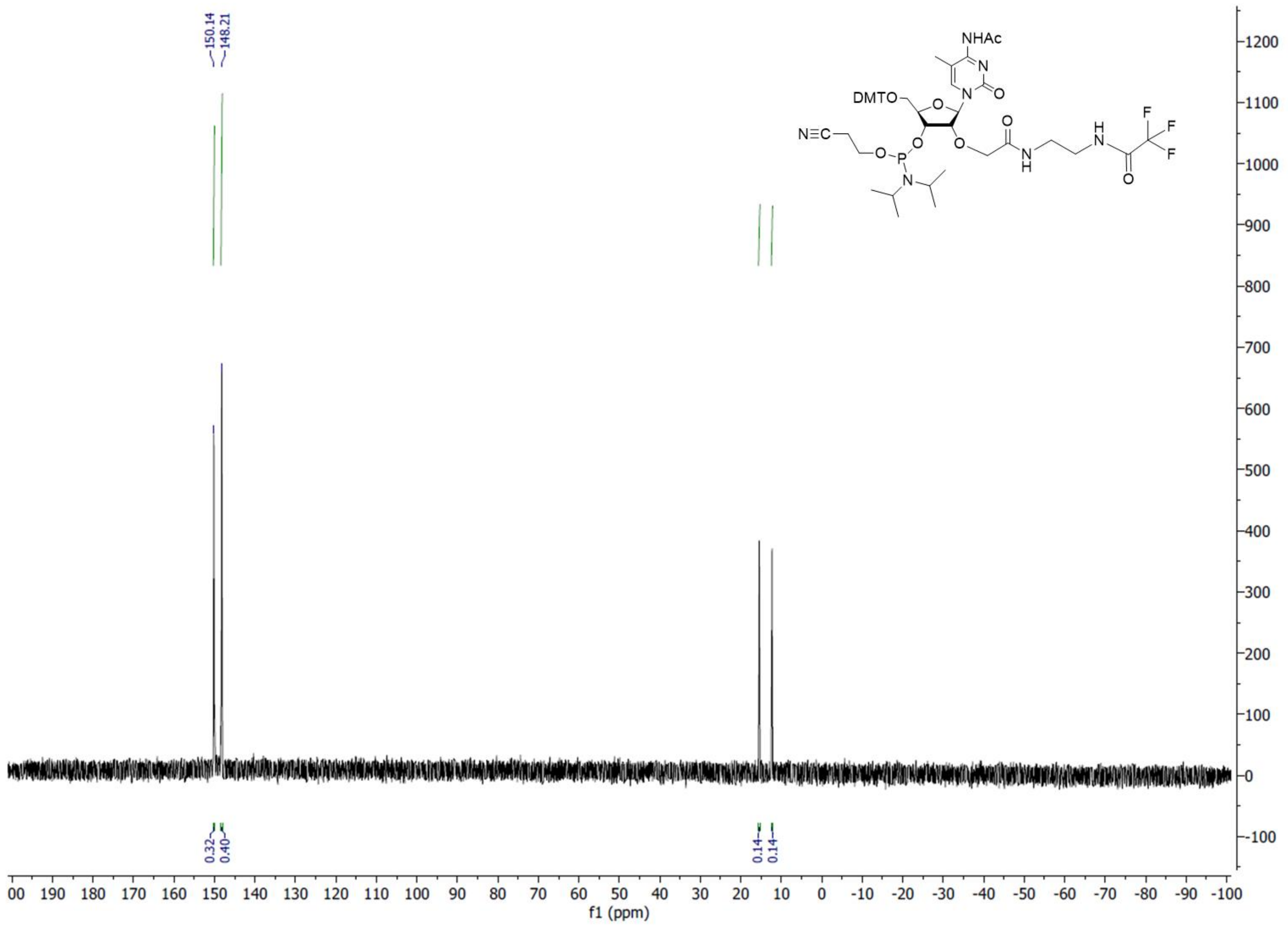

*Figure S29.^{31}P NMR spectrum of compound **16** (202.47 MHz, CD_3CN) before reversed phase chromatography.*

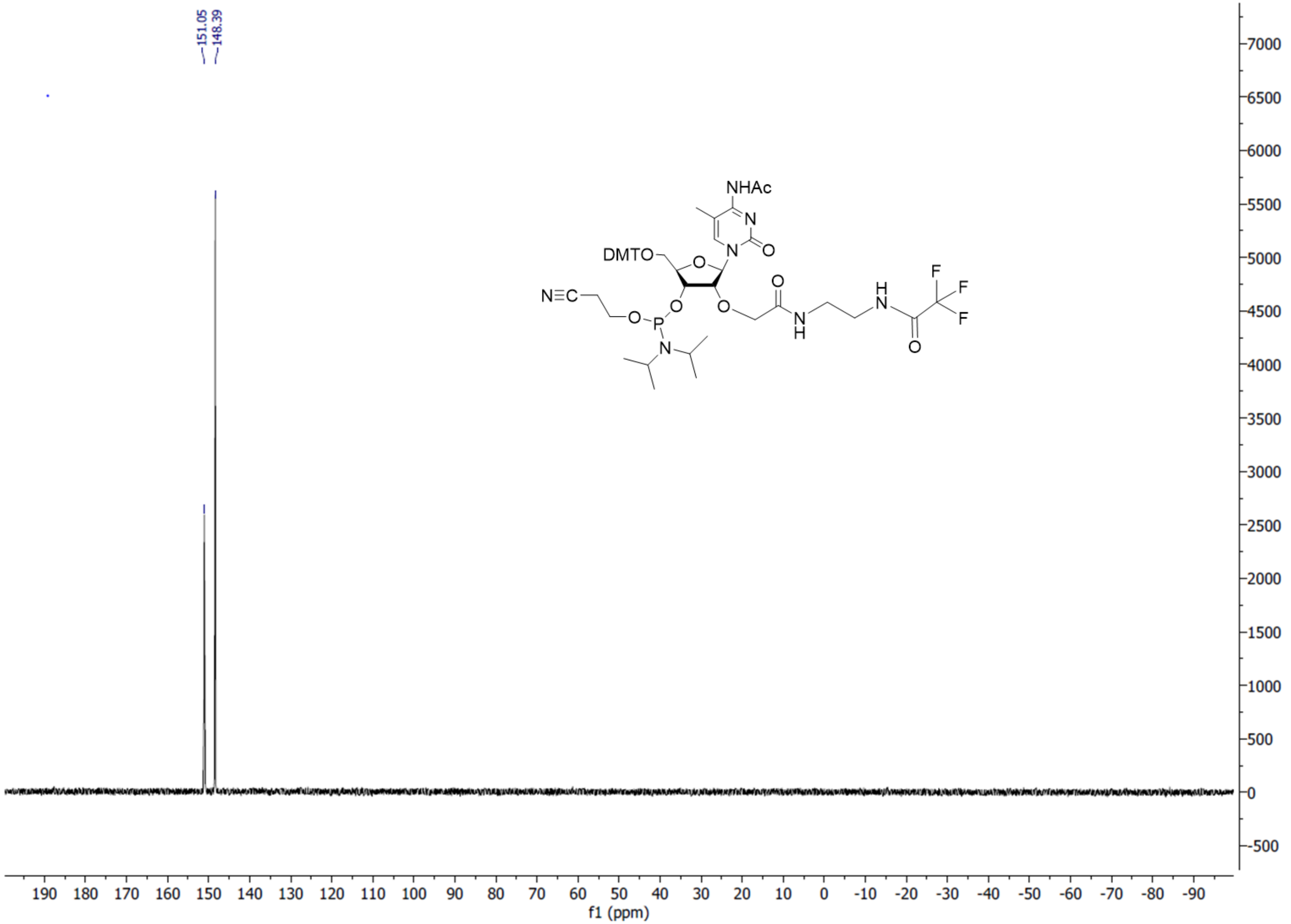

Figure S30.^{31}*P NMR spectrum of compound* ***16*** *(202.47 MHz,* CD_3CN*) after reversed phase chromatography.*

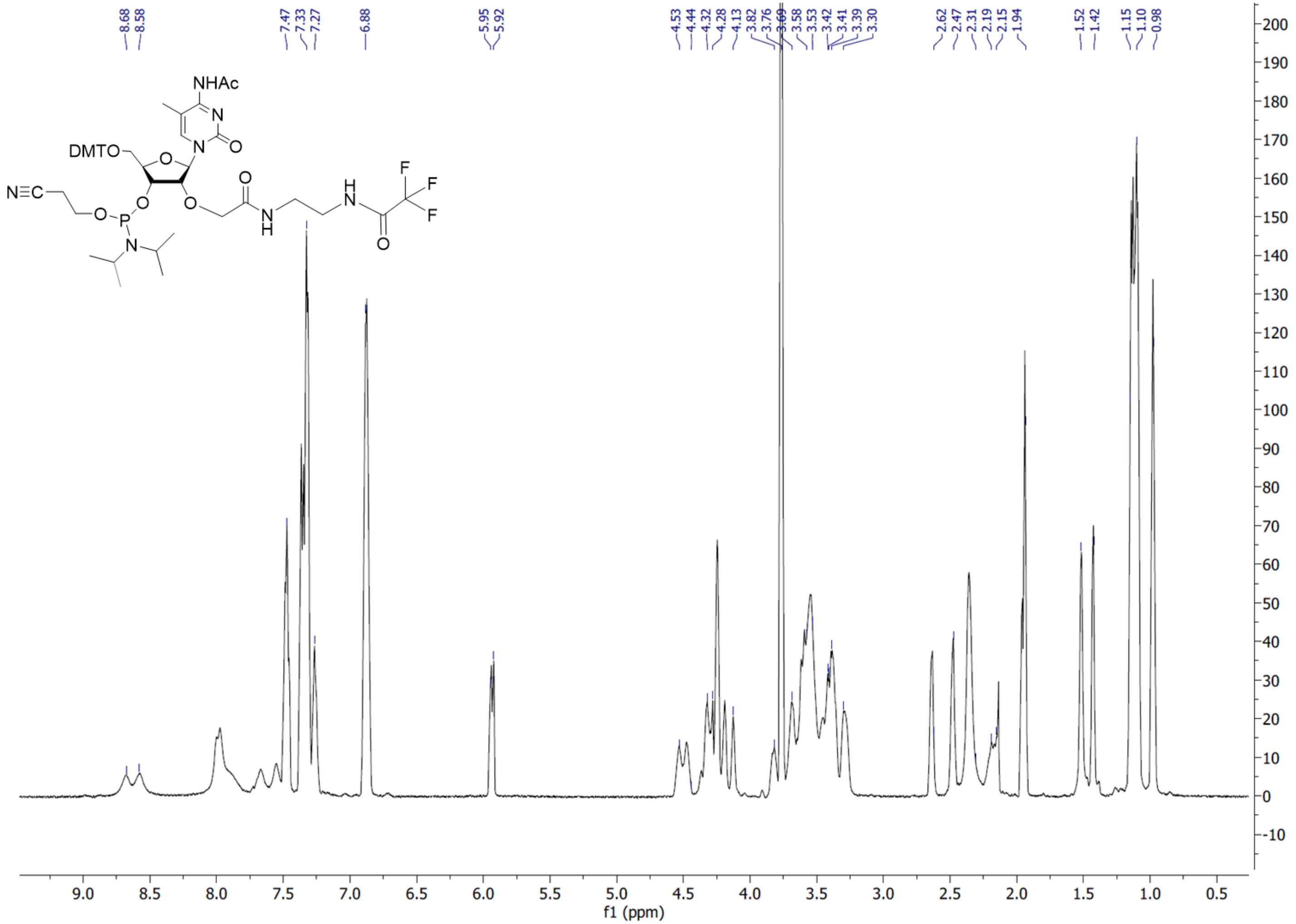

*Figure S31. ^{1}H NMR spectrum of compound **16** (500 MHz, CD_3CN) after reversed phase chromatography.*

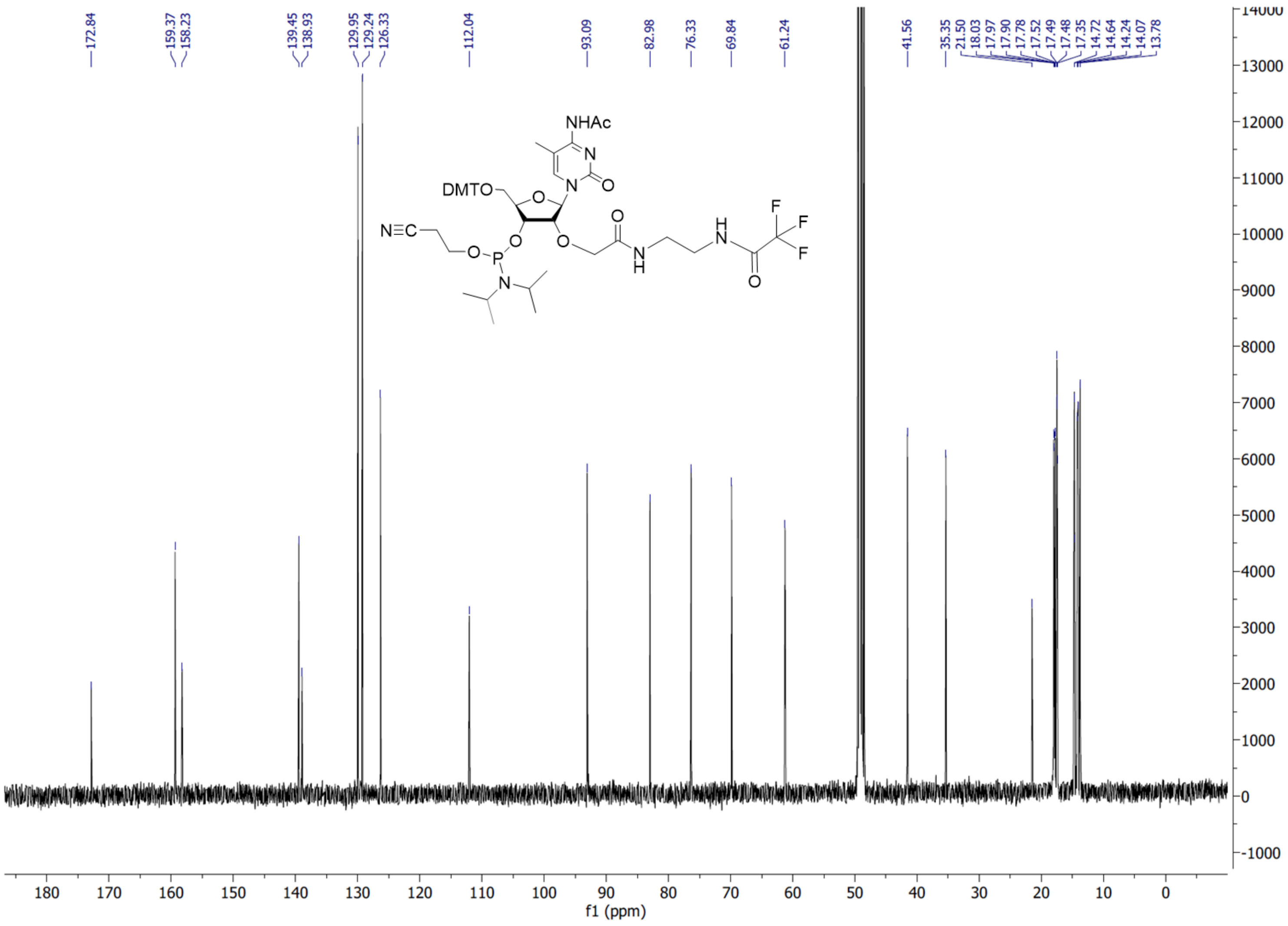

*Figure S32. ^{13}C NMR spectrum of compound **16** (125.76 MHz, CD_3CN) after reversed phase chromatography.*

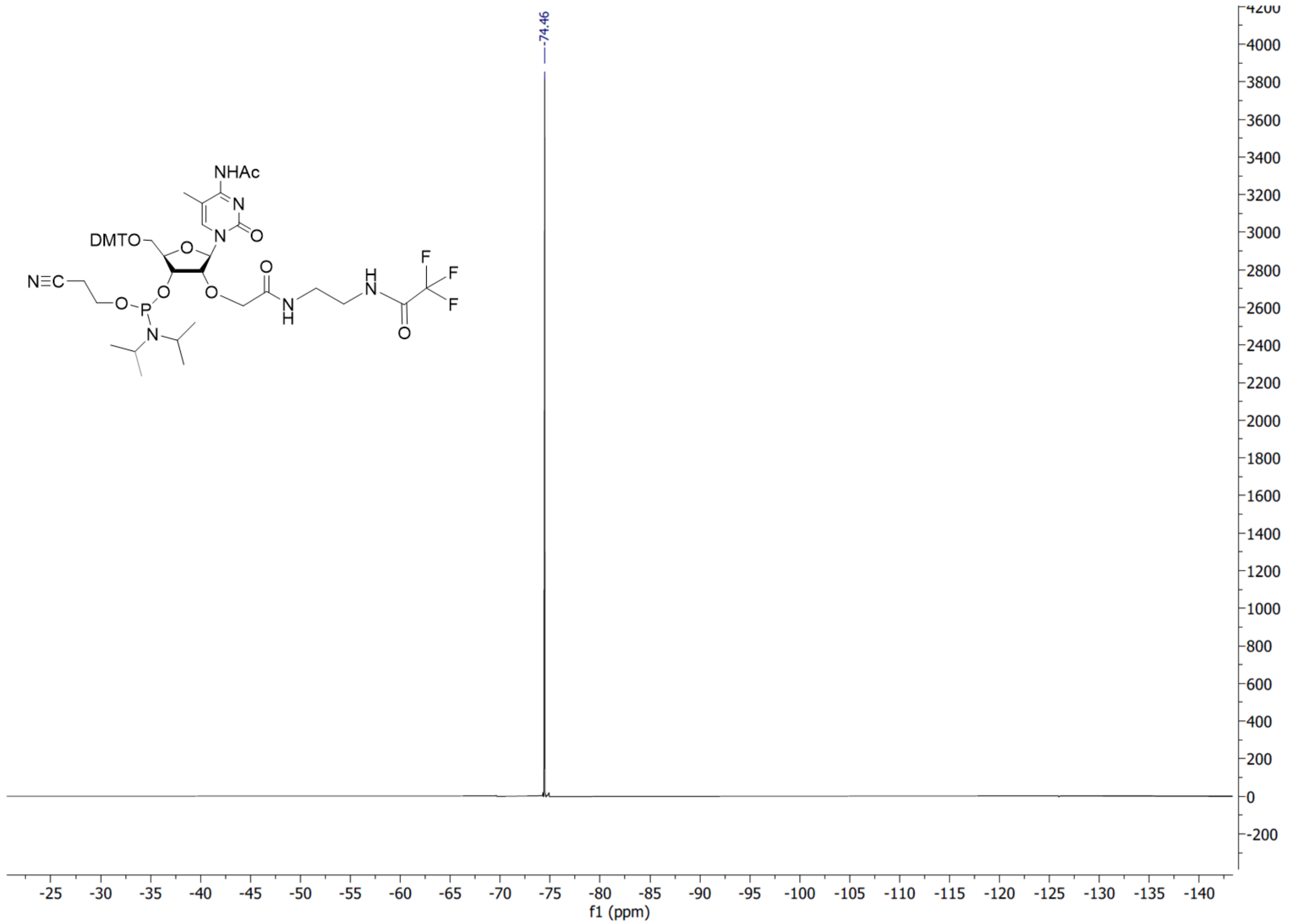

*Figure S33. ^{19}F NMR spectrum of compound **16** (470.56 MHz, DMSO-d$_6$) after reversed phase chromatography.*

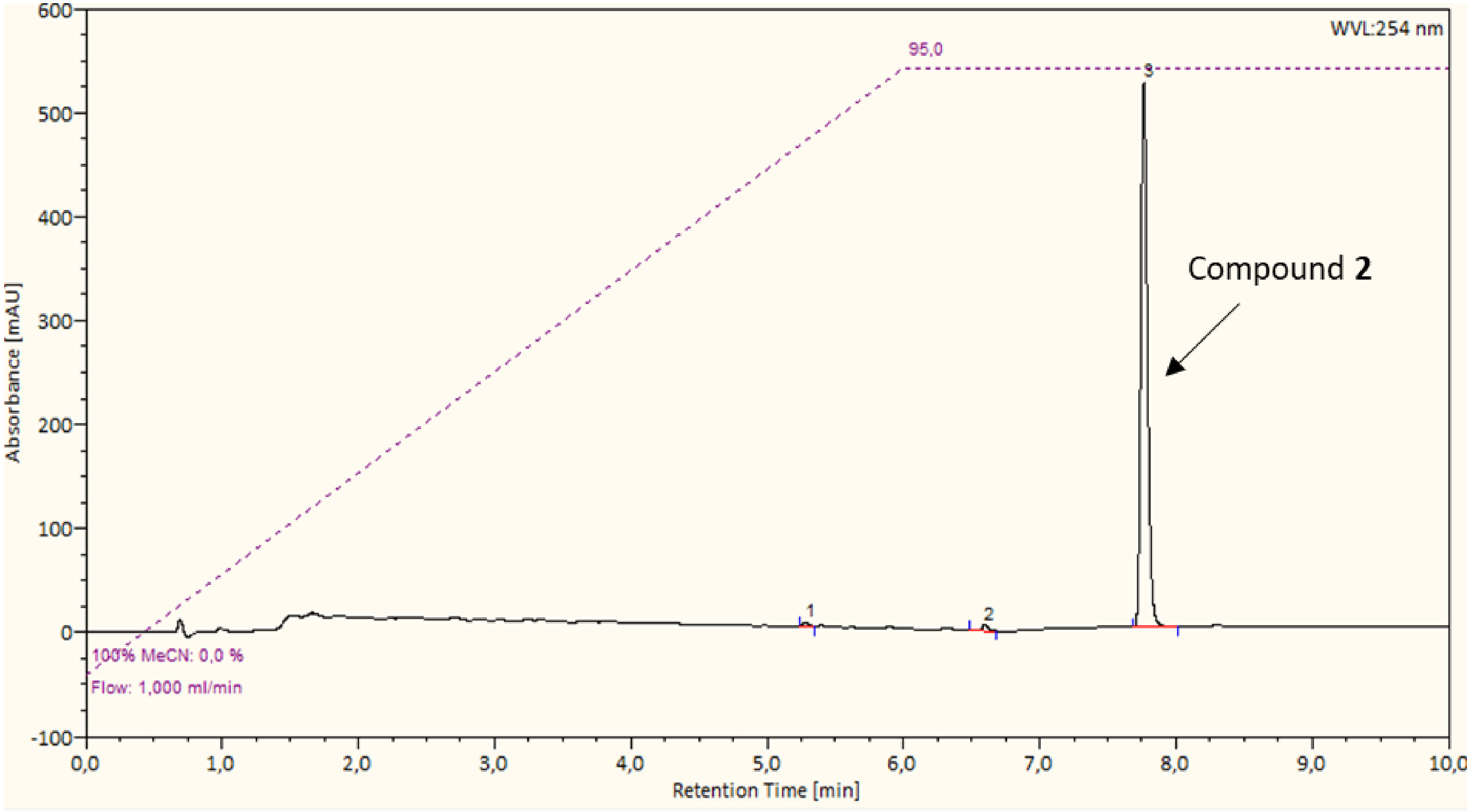

Figure S34 RP-HPLC profile of compound **2** *using RP HPLC on C18 column (Waters XBridge® Oligonucleotide BEH C18) with a linear gradient from 0-100% of buffer B in buffer A over 10 min at 40 °C. Buffer A: 5 mM ammonium acetate, buffer B: MeCN.*

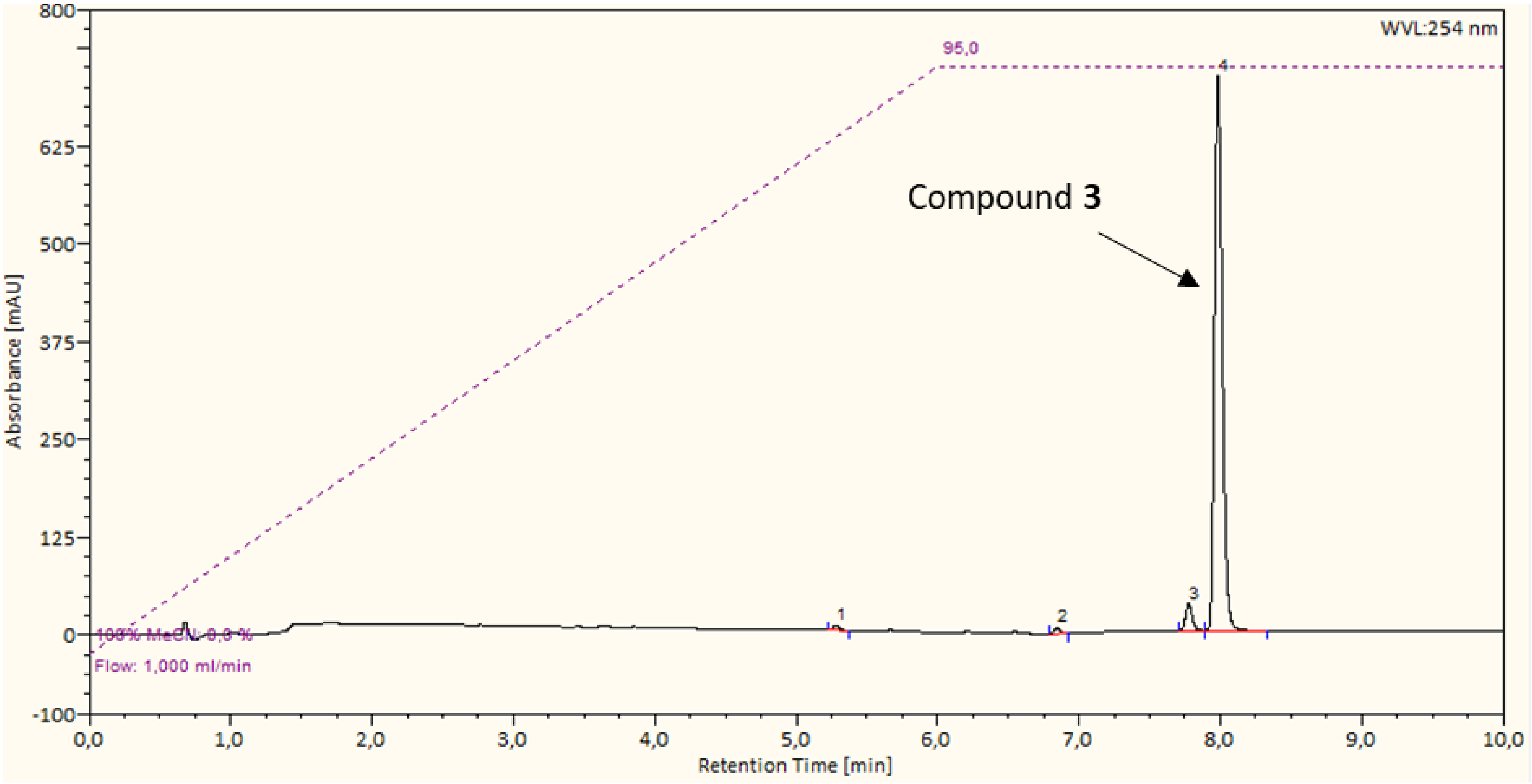

*Figure S35. RP-HPLC profile of compound **3** using RP HPLC on C18 column (Waters XBridge® Oligonucleotide BEH C18) with a linear gradient from 0-100% of buffer B in buffer A over 10 min at 40 °C. Buffer A: 5 mM ammonium acetate, buffer B: MeCN.*

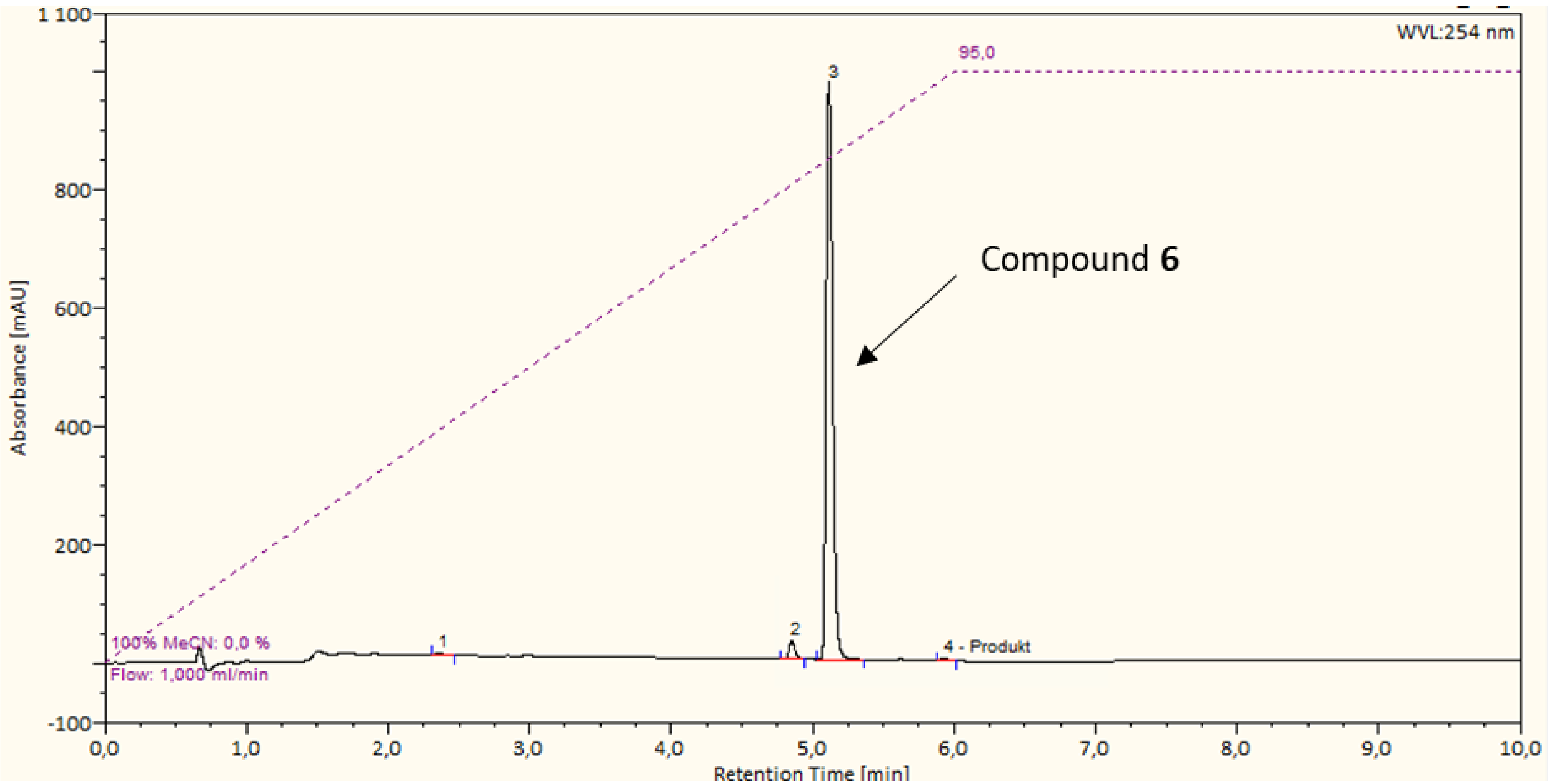

*Figure S36. RP-HPLC profile of compound **6** using RP HPLC on C18 column (Waters XBridge® Oligonucleotide BEH C18) with a linear gradient from 0-100% of buffer B in buffer A over 10 min at 40 °C. Buffer A: 5 mM ammonium acetate, buffer B: MeCN.*

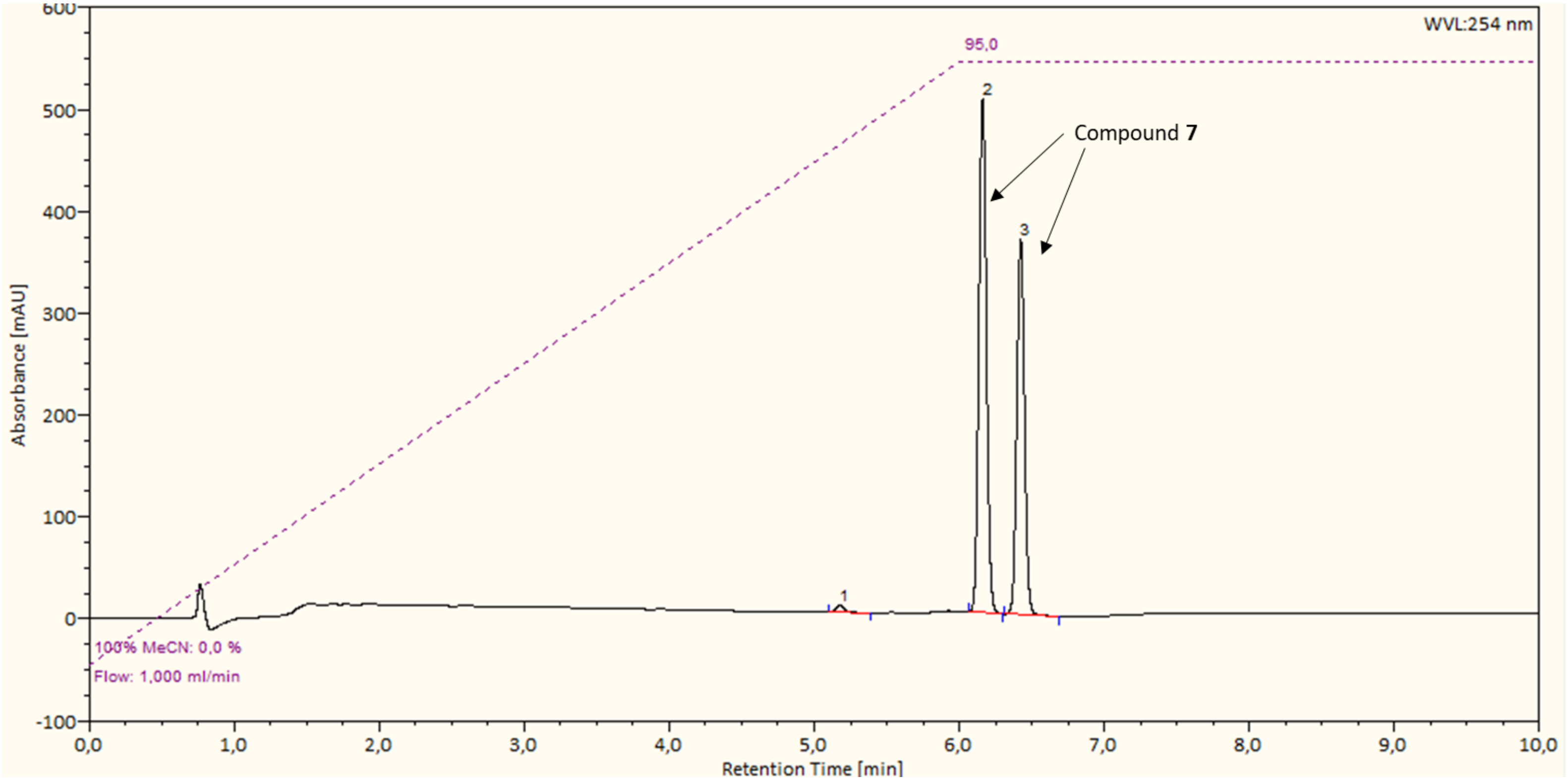

Figure S37. RP-HPLC profile of compound **7** *using RP HPLC on C18 column (Waters XBridge® Oligonucleotide BEH C18) with a linear gradient from 0-100% of buffer B in buffer A over 10 min at 40 °C. Buffer A: 5 mM ammonium acetate, buffer B: MeCN.*

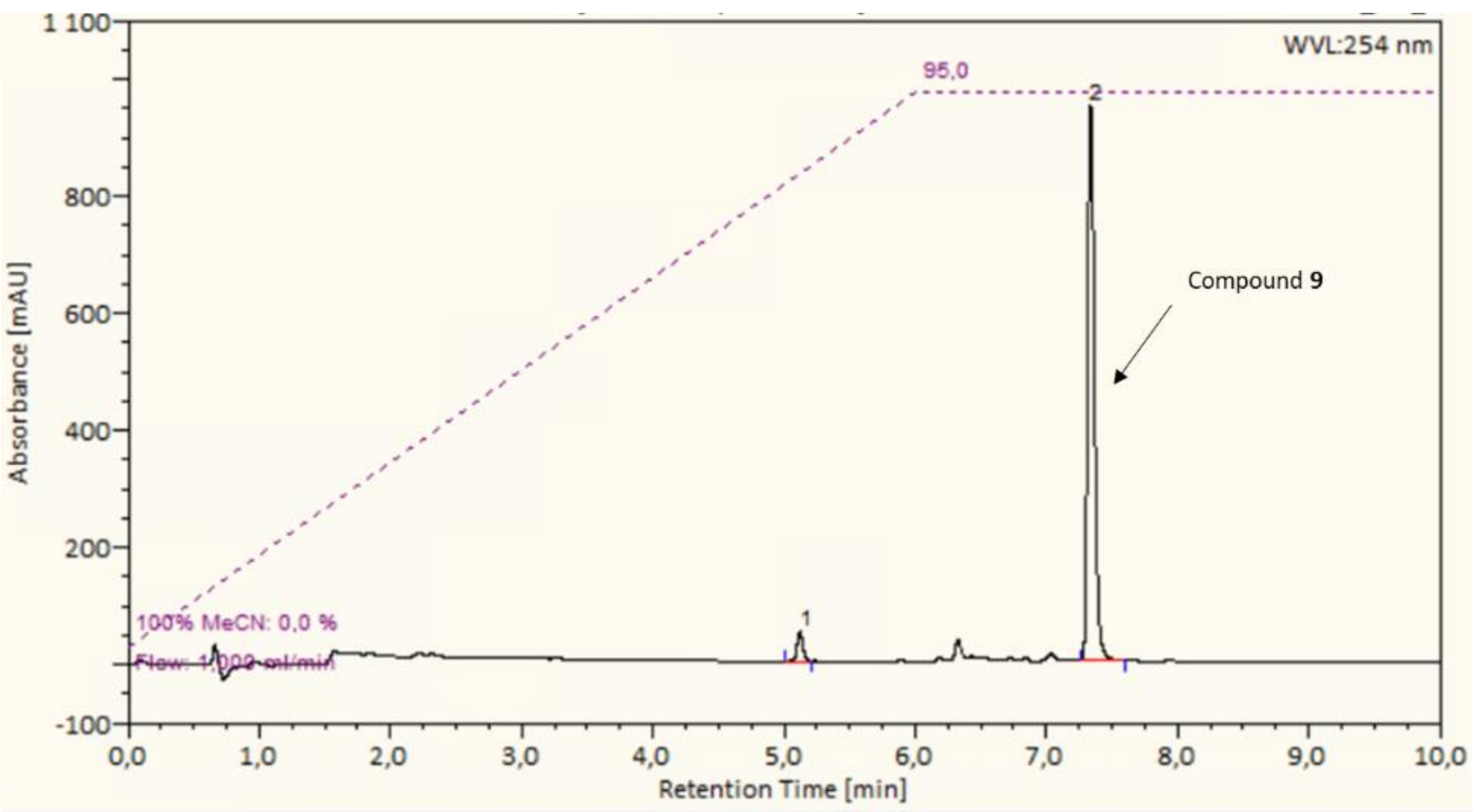

*Figure S38. RP-HPLC profile of compound **9** using RP HPLC on C18 column (Waters XBridge® Oligonucleotide BEH C18) with a linear gradient from 0-100% of buffer B in buffer A over 10 min at 40 °C. Buffer A: 5 mM ammonium acetate, buffer B: MeCN.*

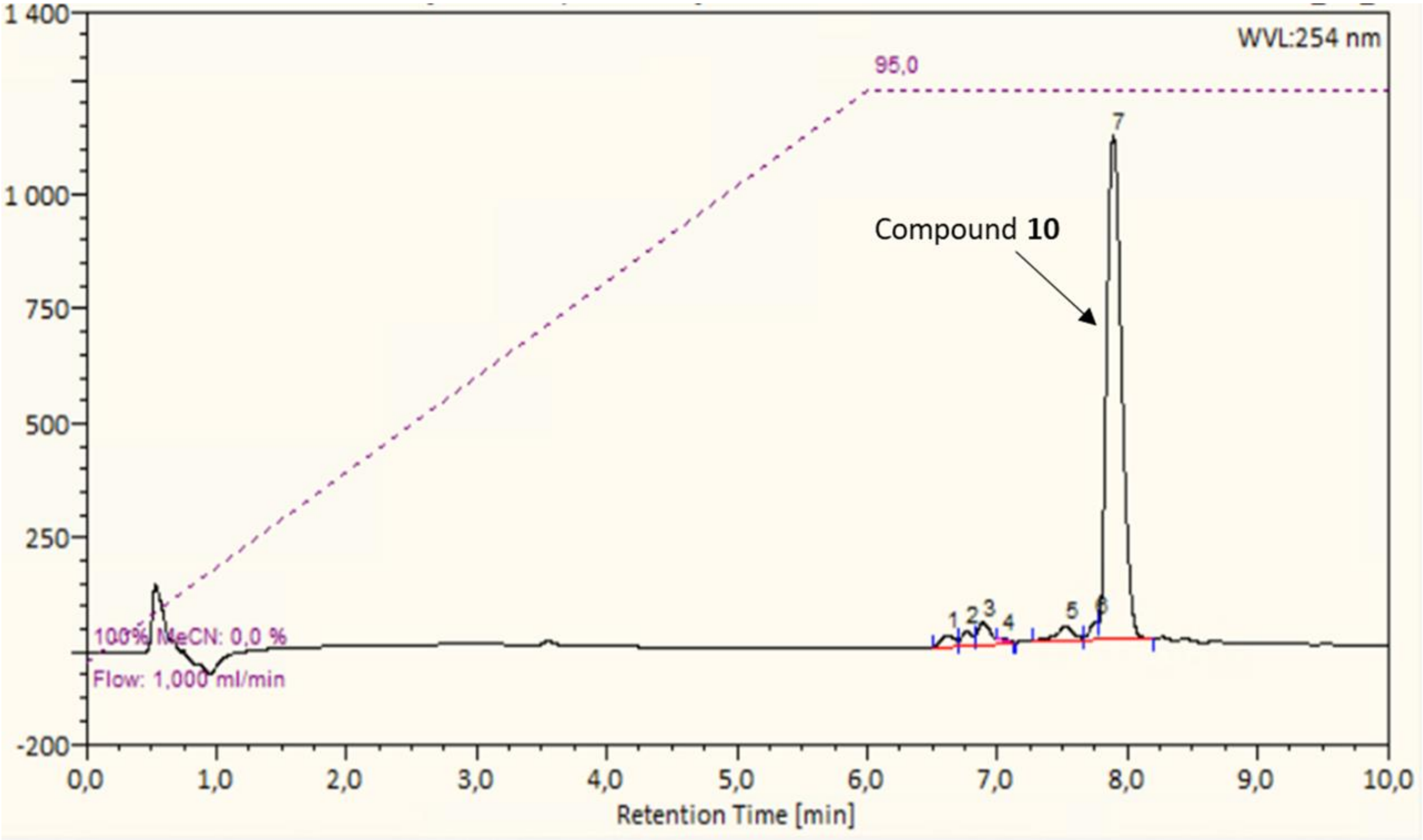

Figure S39. RP-HPLC profile of compound ***10*** *using RP HPLC on C18 column (Waters XBridge® Oligonucleotide BEH C18) with a linear gradient from 0-100% of buffer B in buffer A over 10 min at 40 °C. Buffer A: 5 mM ammonium acetate, buffer B: MeCN.*

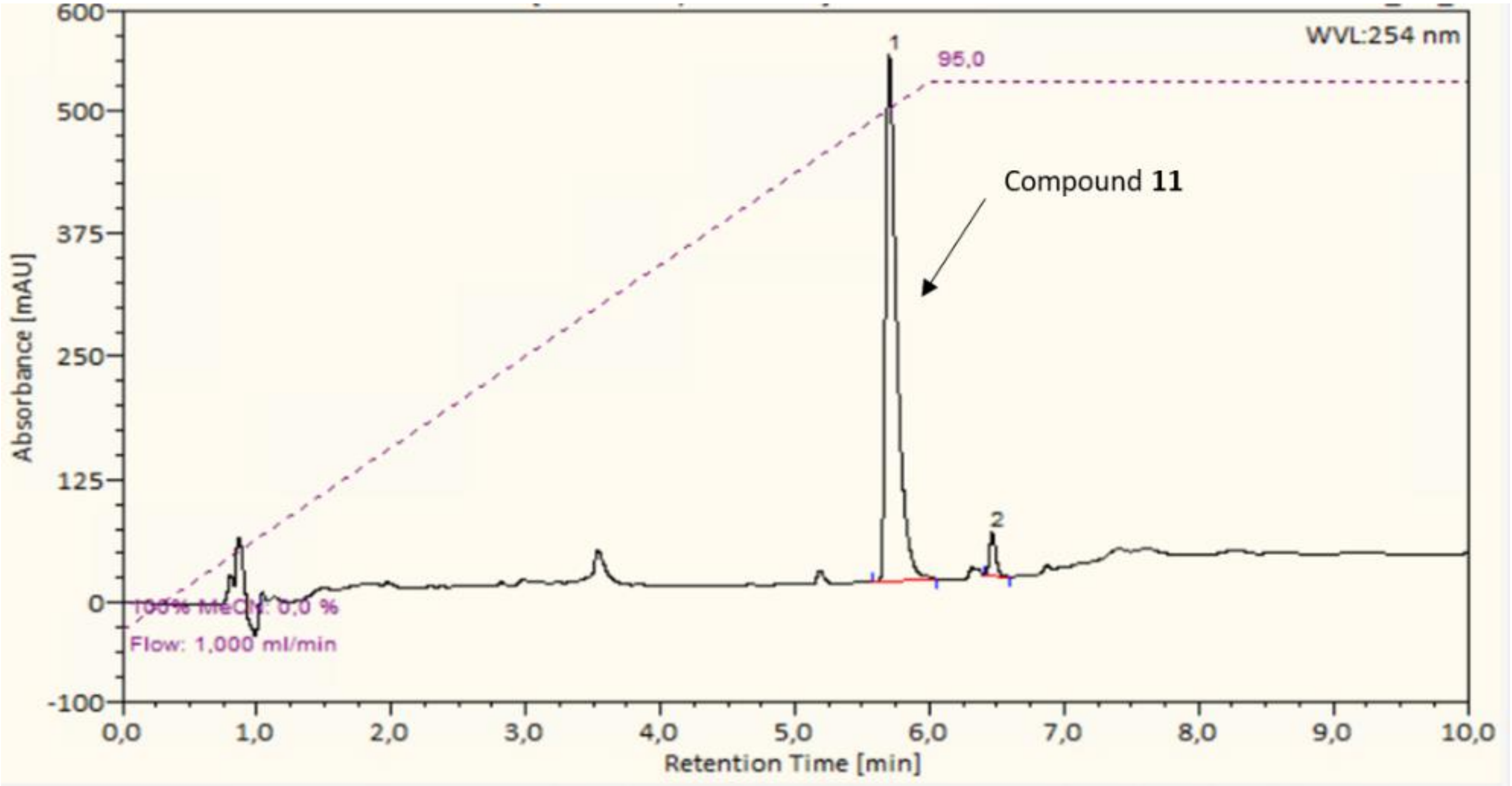

*Figure S40. RP-HPLC profile of compound **11** using RP HPLC on C18 column (Waters XBridge® Oligonucleotide BEH C18) with a linear gradient from 0-100% of buffer B in buffer A over 10 min at 40 °C. Buffer A: 5 mM ammonium acetate, buffer B: MeCN.*

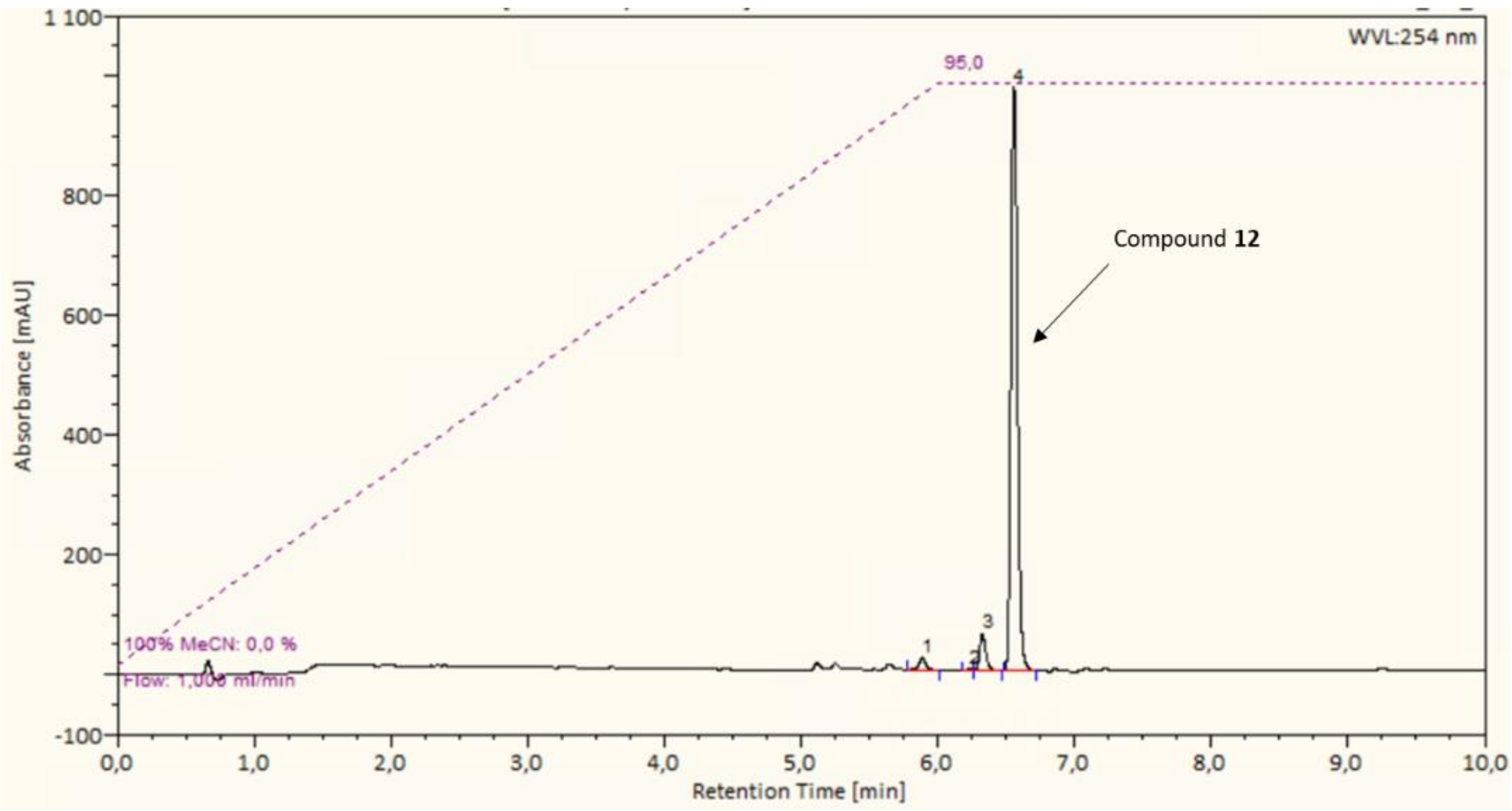

*Figure S41. RP-HPLC profile of compound **12** using RP HPLC on C18 column (Waters XBridge® Oligonucleotide BEH C18) with a linear gradient from 0-100% of buffer B in buffer A over 10 min at 40 °C. Buffer A: 5 mM ammonium acetate, buffer B: MeCN.*

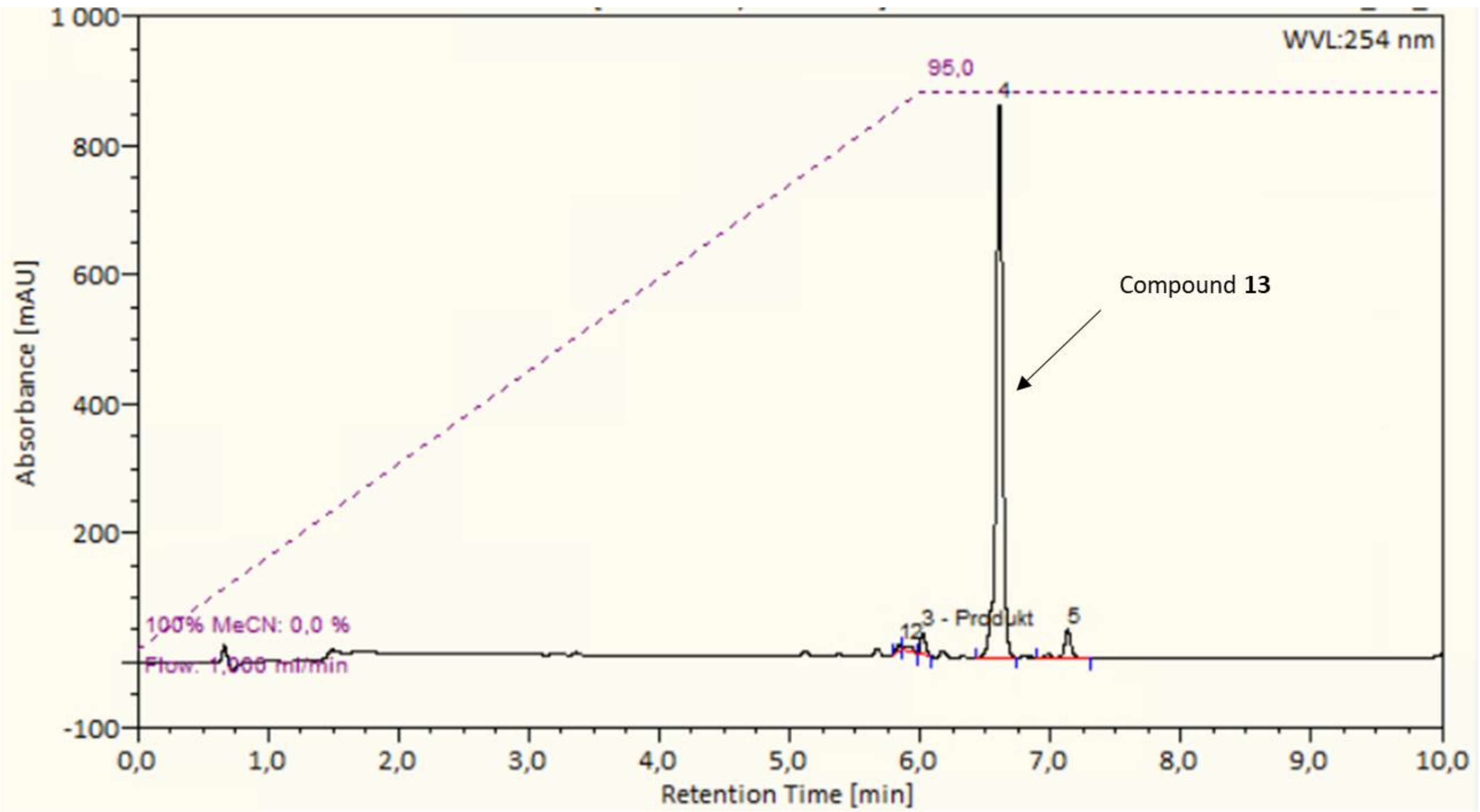

*Figure S42. RP-HPLC profile of compound **13** using RP HPLC on C18 column (Waters XBridge® Oligonucleotide BEH C18) with a linear gradient from 0-100% of buffer B in buffer A over 10 min at 40 °C. Buffer A: 5 mM ammonium acetate, buffer B: MeCN.*

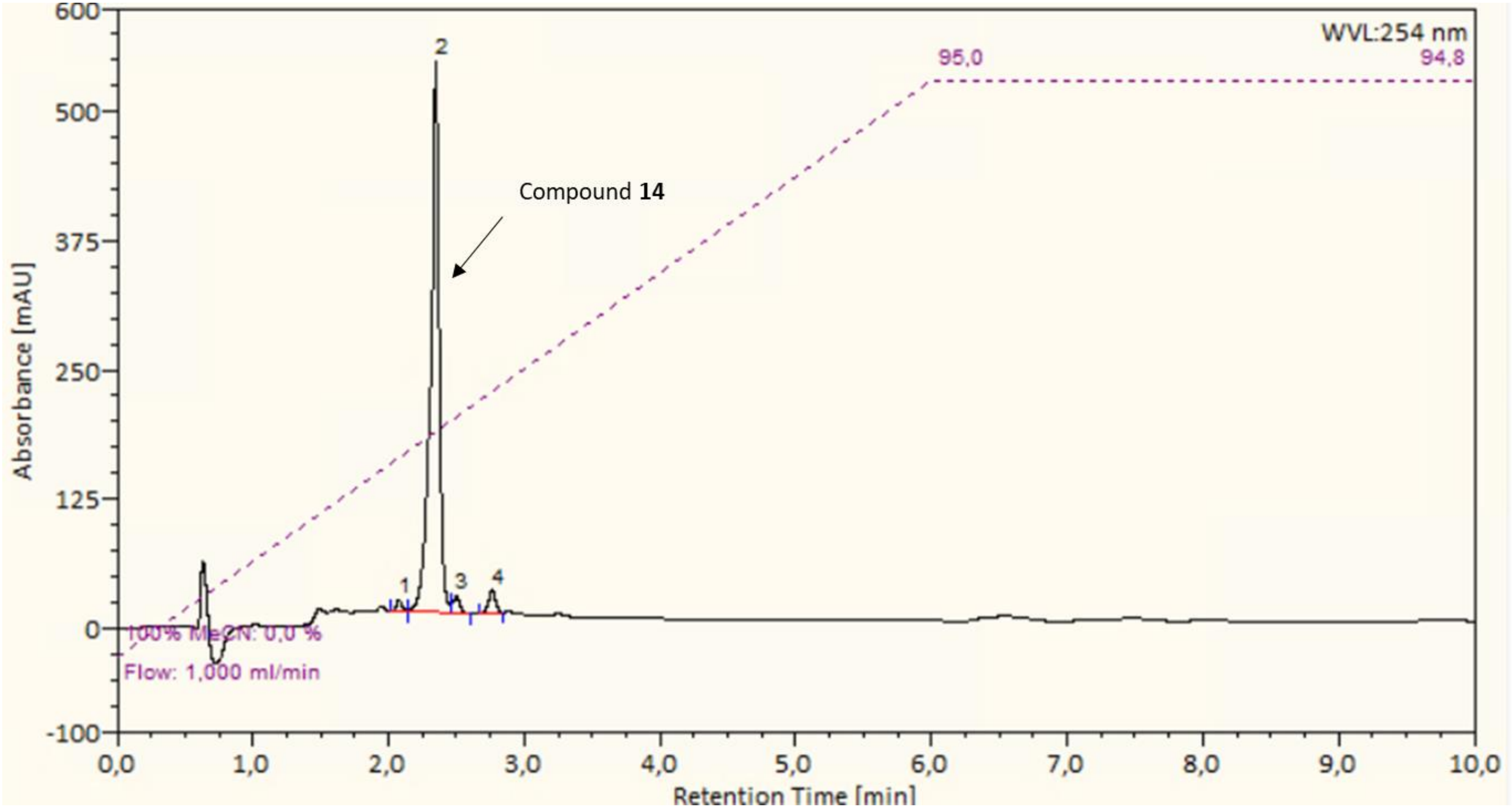

*Figure S43. RP-HPLC profile of compound **14** using RP HPLC on C18 column (Waters XBridge® Oligonucleotide BEH C18) with a linear gradient from 0-100% of buffer B in buffer A over 10 min at 40 °C. Buffer A: 5 mM ammonium acetate, buffer B: MeCN.*

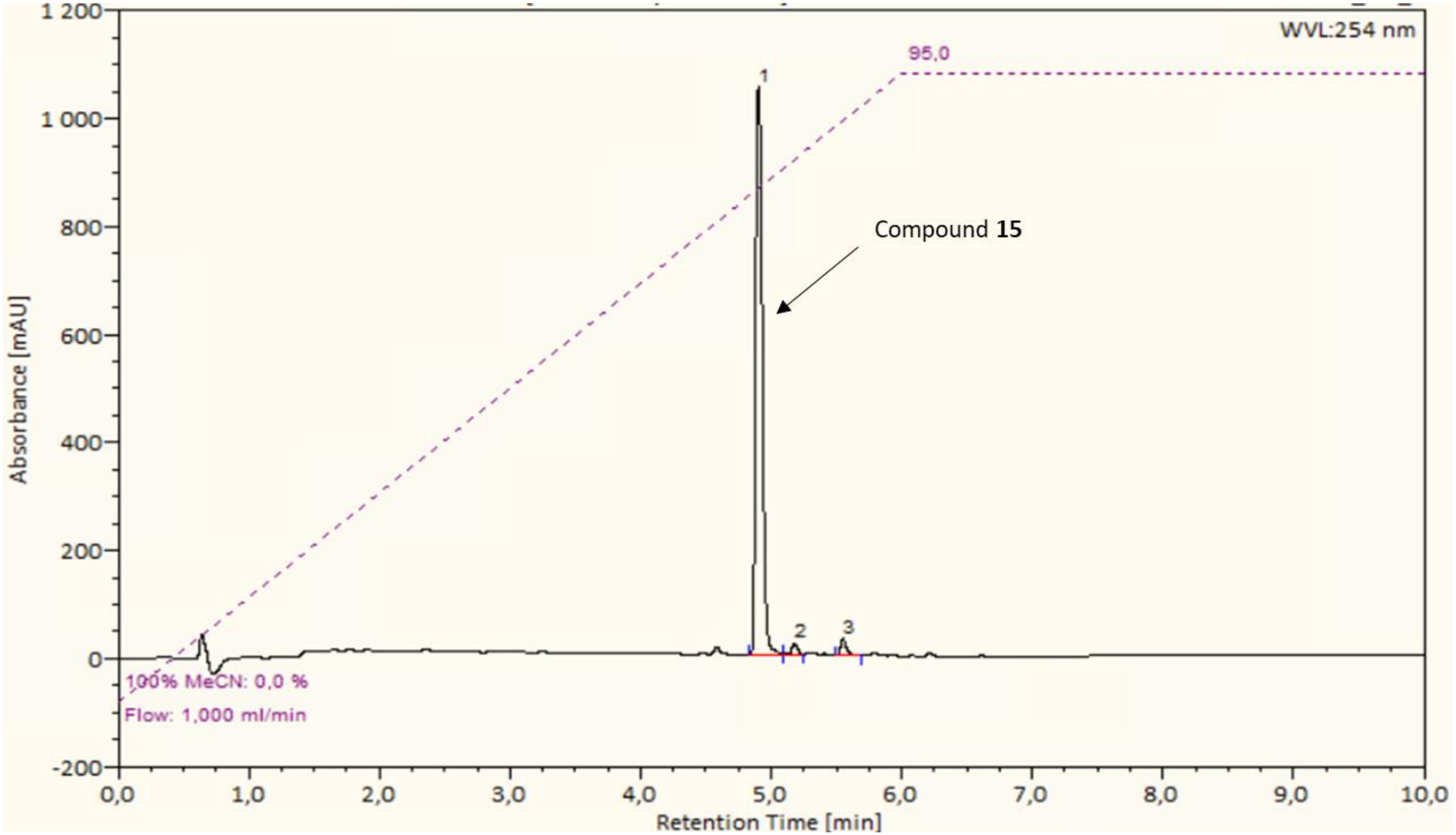

*Figure S44. RP-HPLC profile of compound **15** using RP HPLC on C18 column (Waters XBridge® Oligonucleotide BEH C18) with a linear gradient from 0-100% of buffer B in buffer A over 10 min at 40 °C. Buffer A: 5 mM ammonium acetate, buffer B: MeCN.*

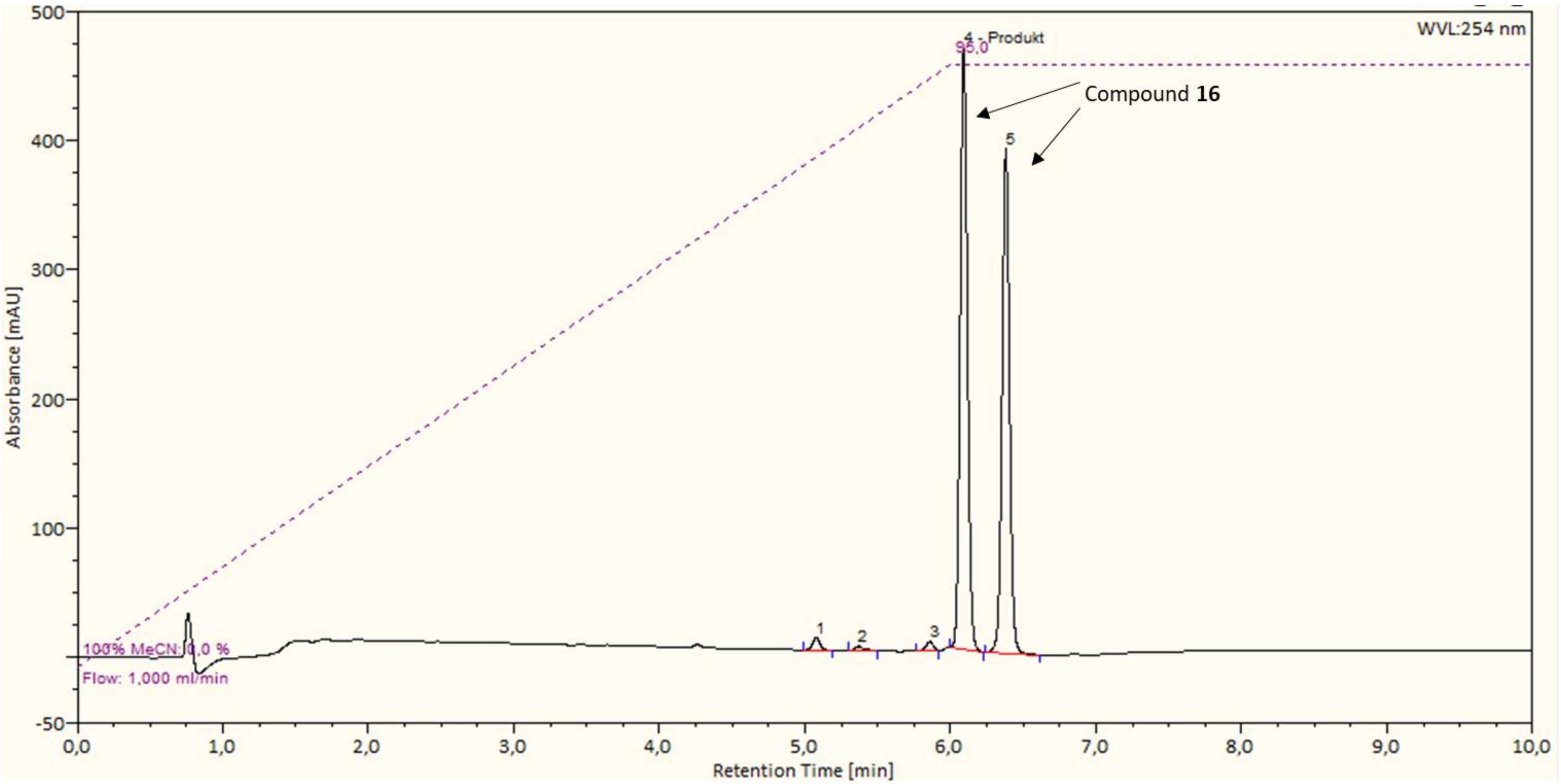

Figure S45. RP-HPLC profile of compound ***16*** *using RP HPLC on C18 column (Waters XBridge® Oligonucleotide BEH C18) with a linear gradient from 0-100% of buffer B in buffer A over 10 min at 40 °C. Buffer A: 5 mM ammonium acetate, buffer B: MeCN*

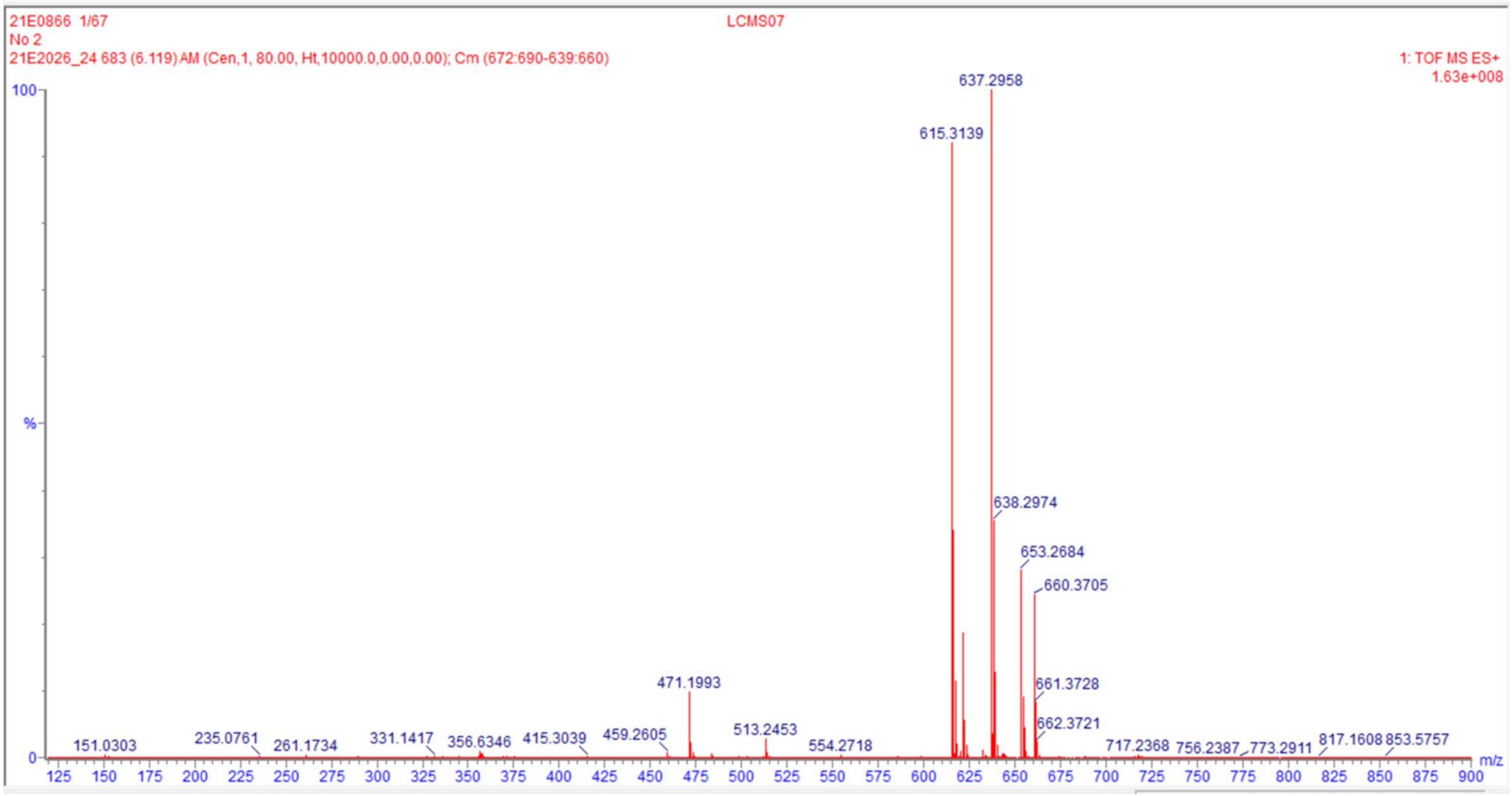

*Figure S46. ESI-TOF mass spectrum of compound **2**. Masses above [MH]+ 615 are adducts.*

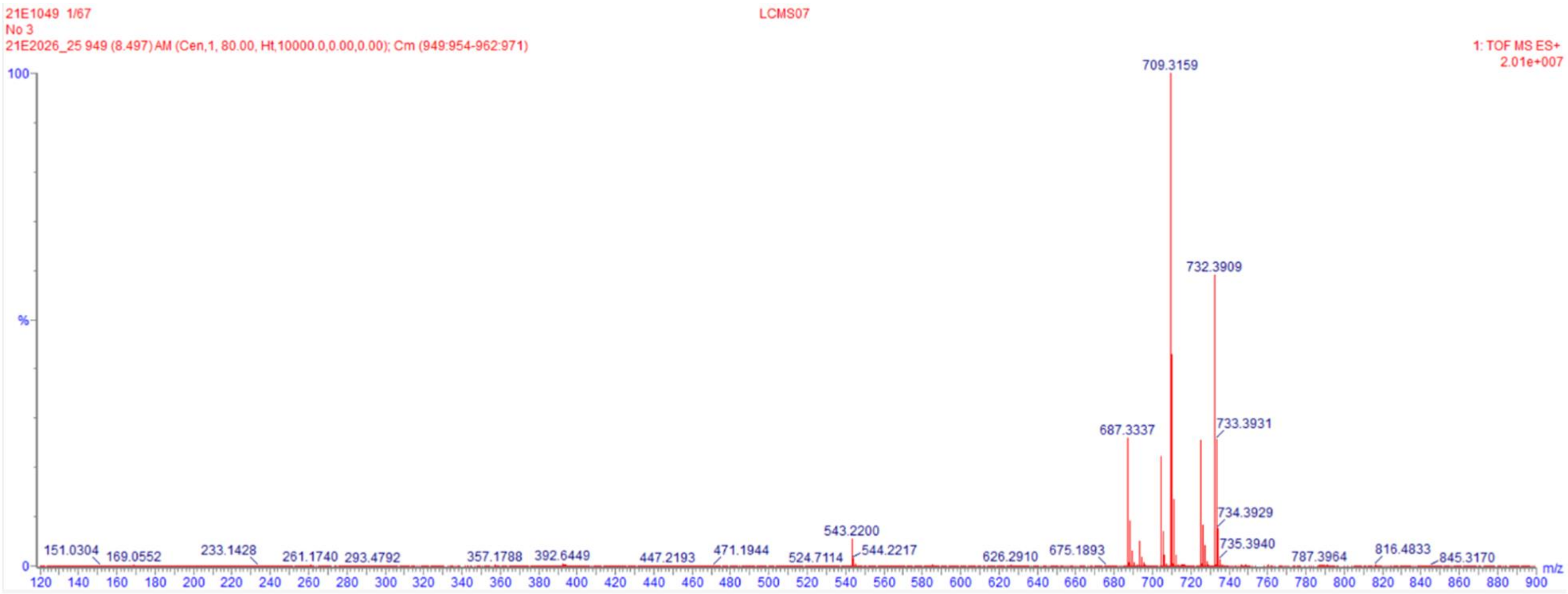

*Figure S47. ESI-TOF mass spectrum of compound **3**. Masses above [MH]+ 687 are adducts.*

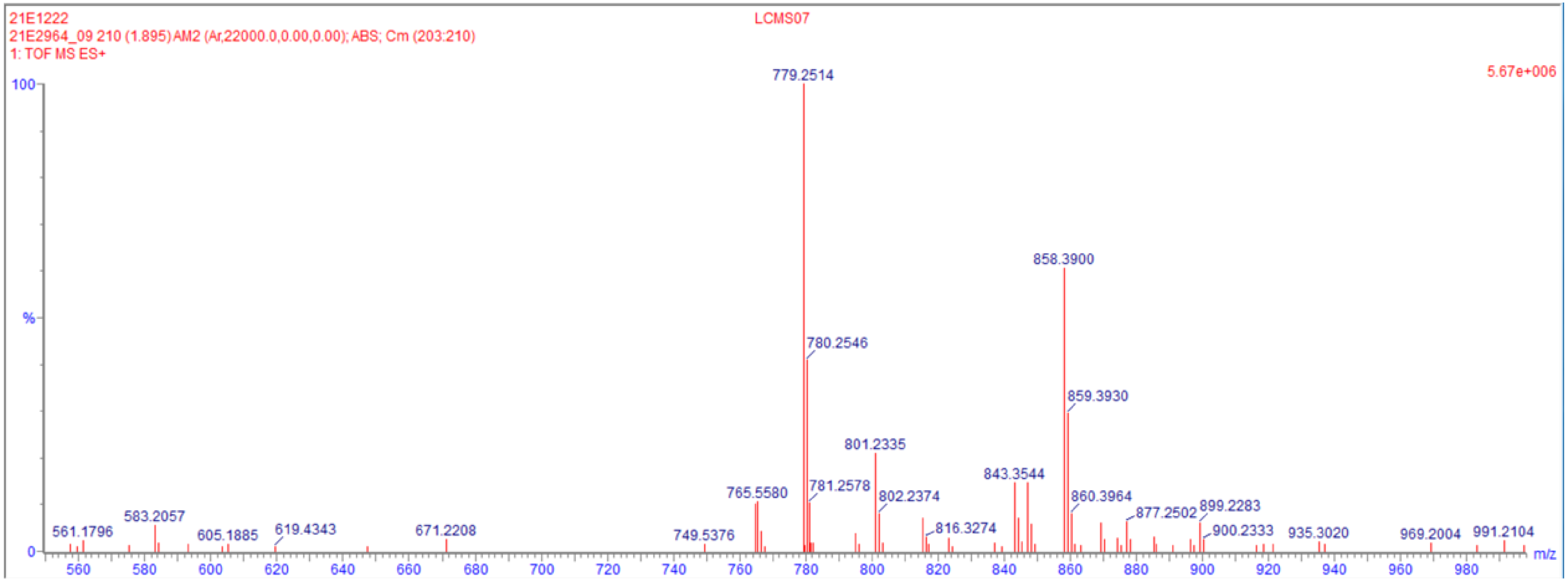

Figure S48. ESI-TOF mass spectrum of compound ***6.*** *M/z 779 = [MNa]+.*

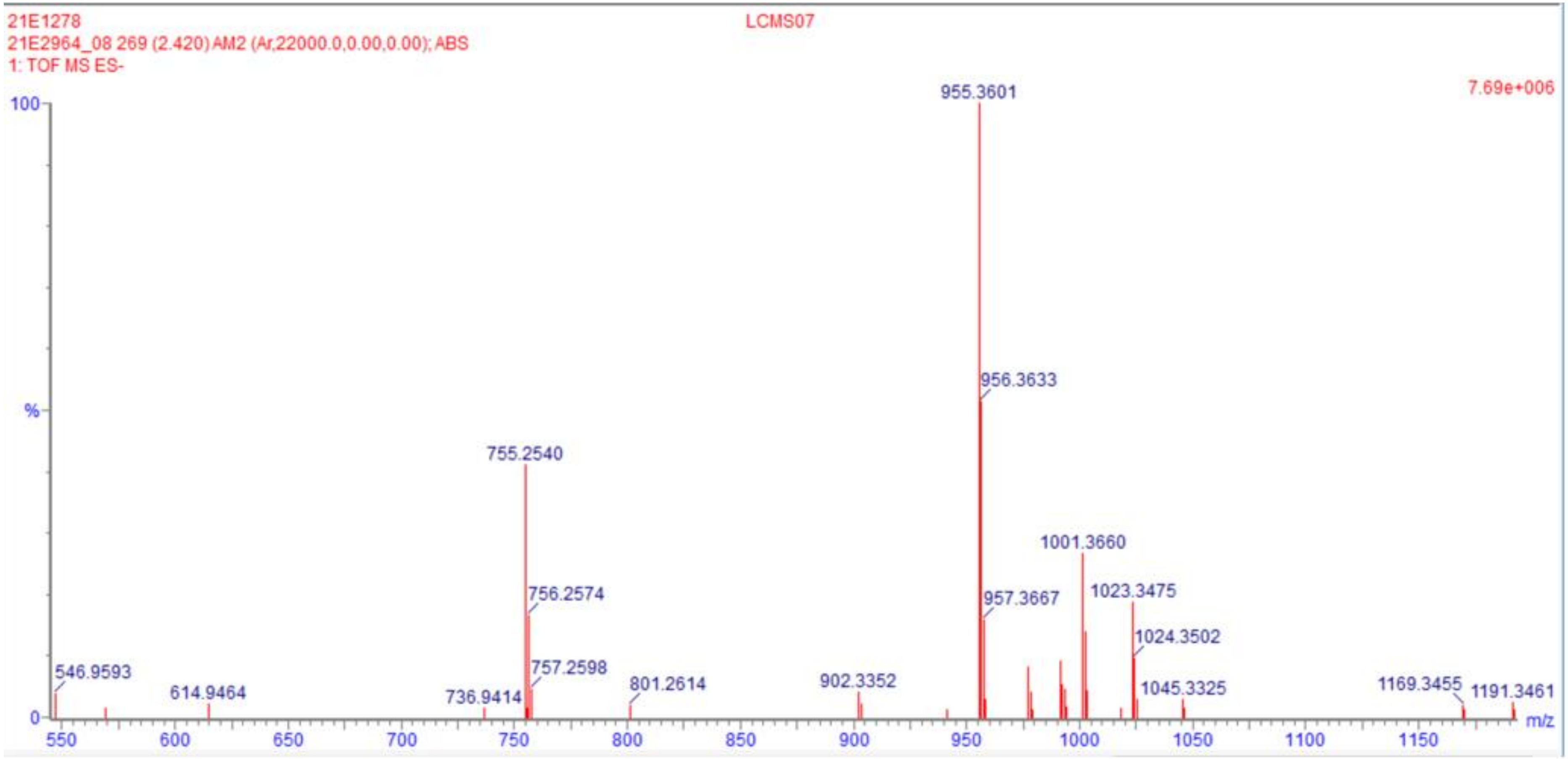

*Figure S49. ESI-TOF mass spectrum of compound **7.***

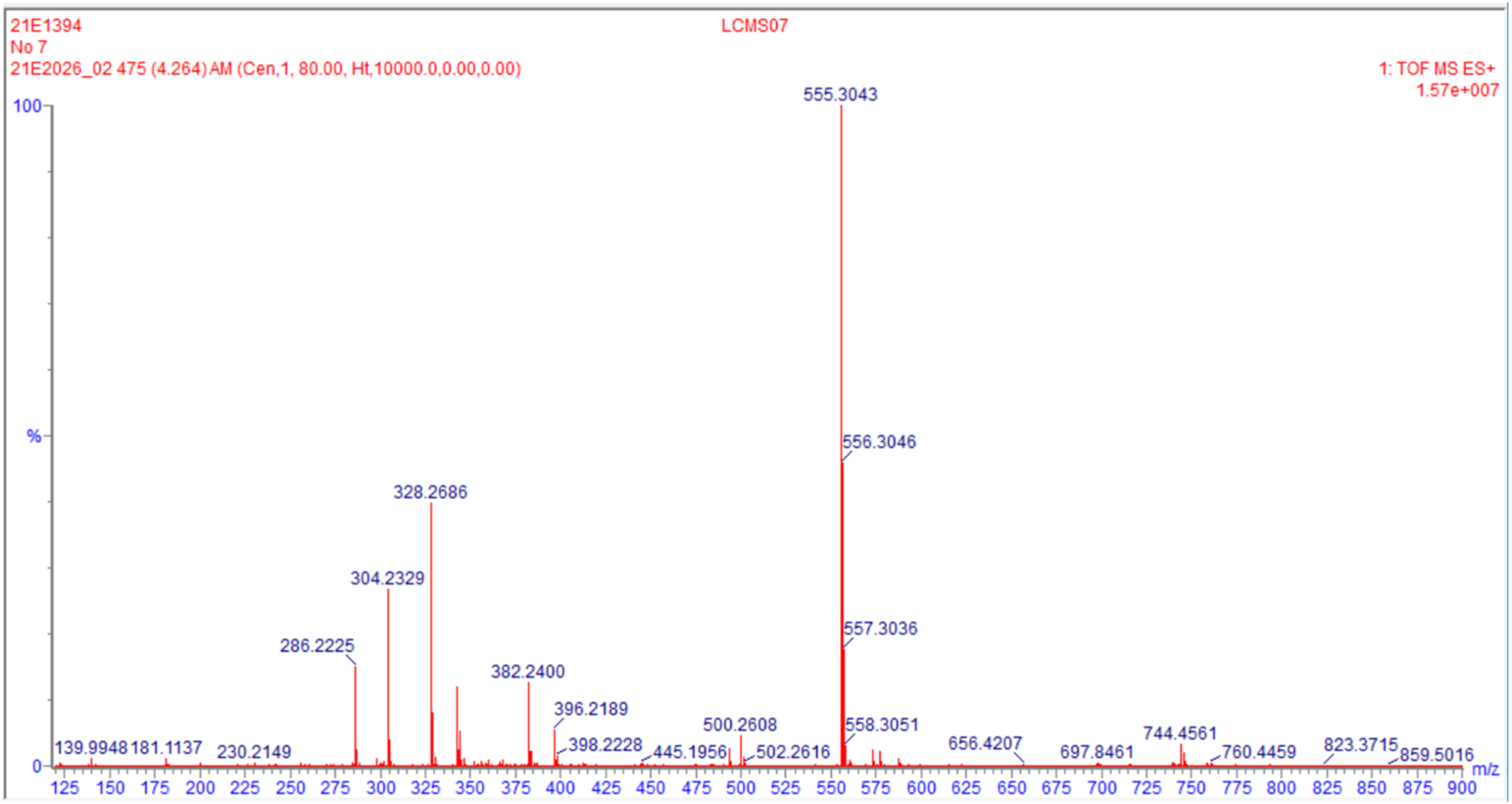

Figure S50. ESI-TOF mass spectrum of compound **9**.

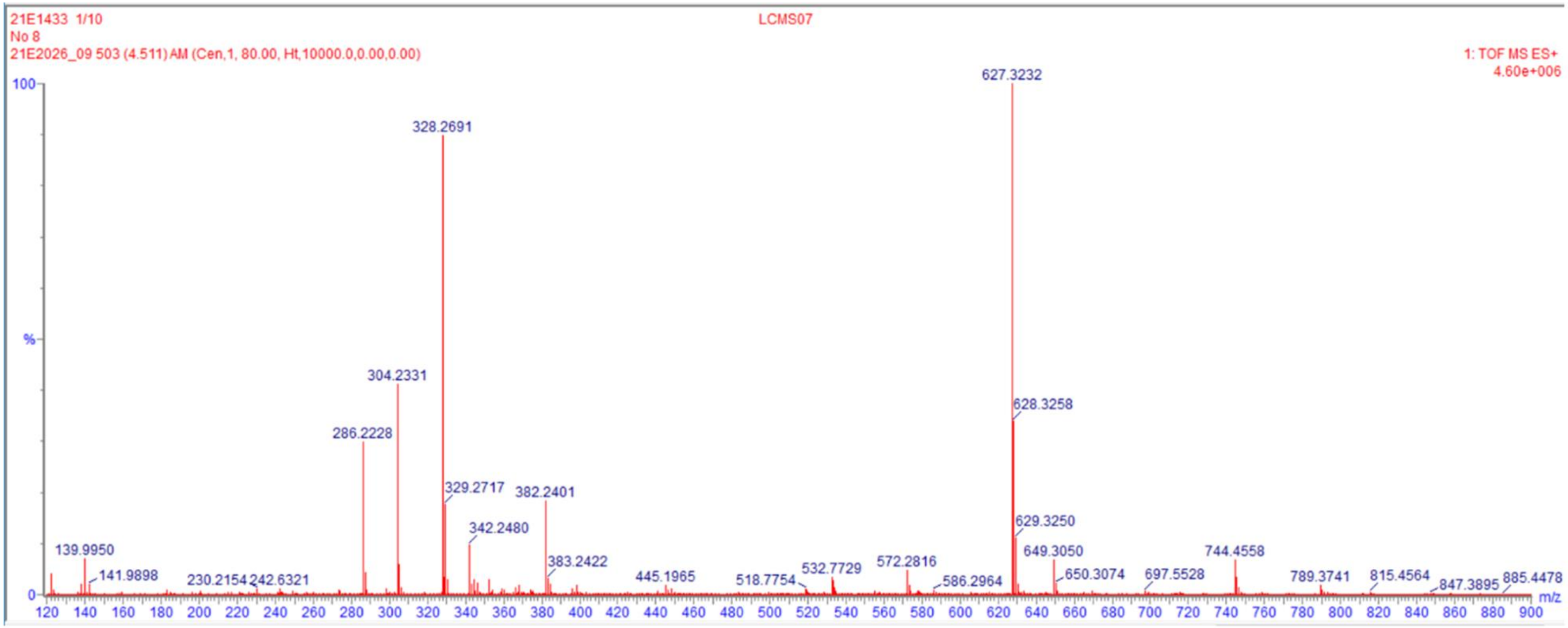

*Figure S51. ESI-TOF mass spectrum of compound **10**.*

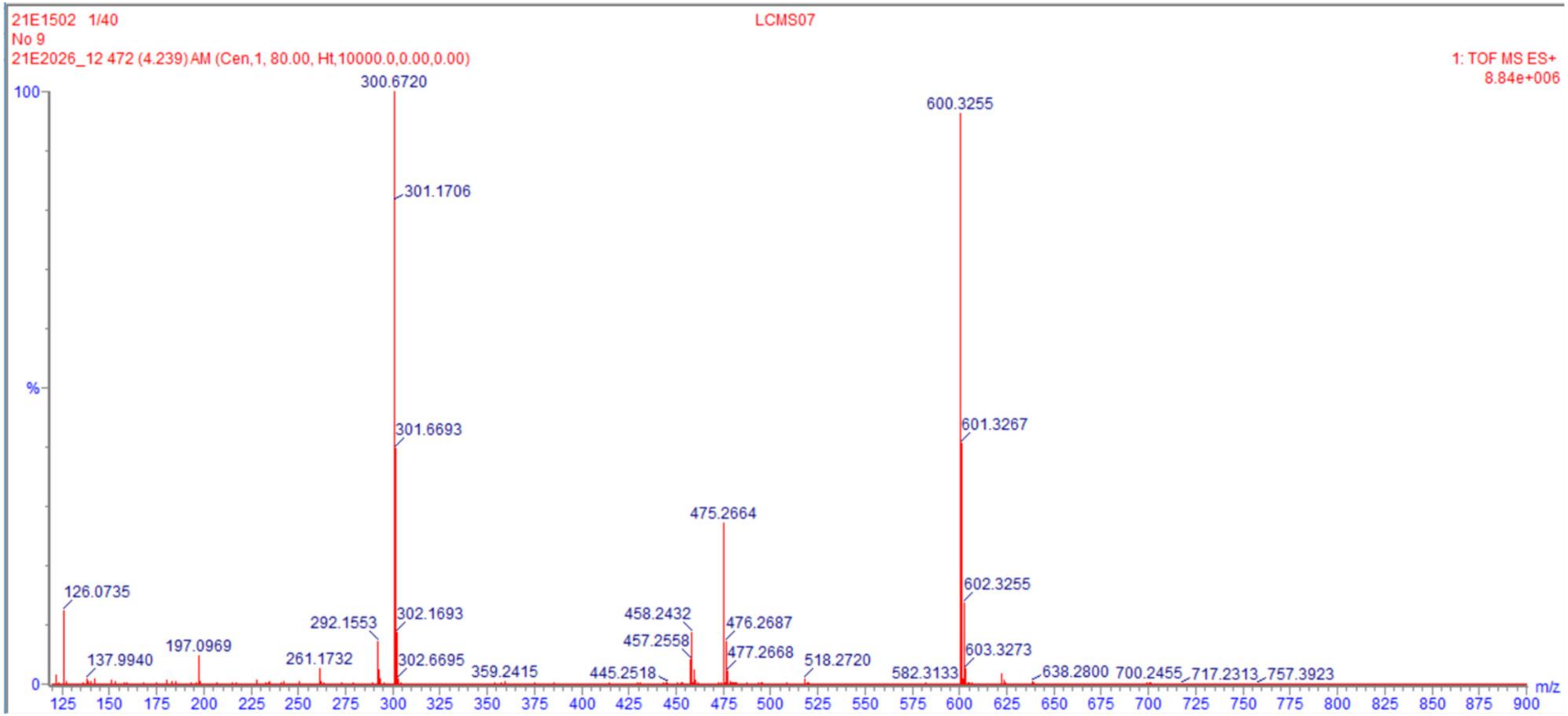

*Figure S52. ESI-TOF mass spectrum of compound **11**. M/z 300 is a fragment of the compound.*

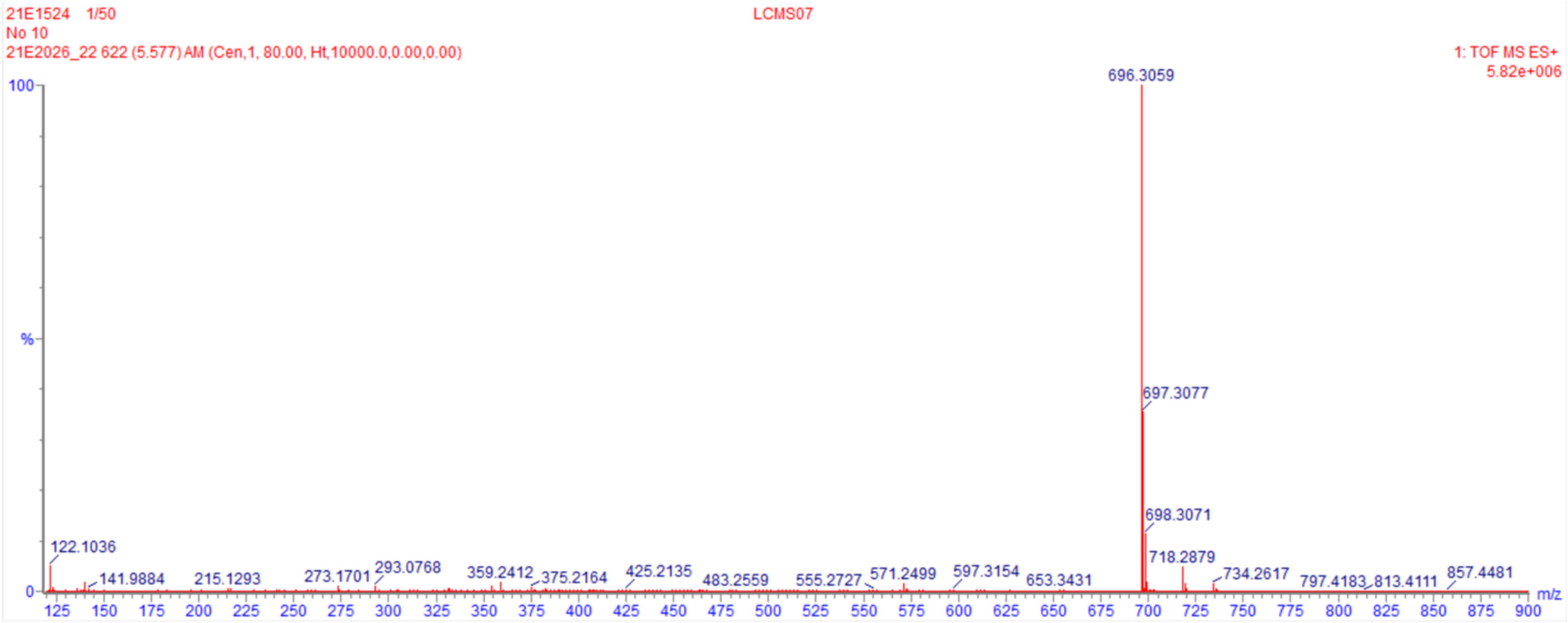

*Figure S53. ESI-TOF mass spectrum of compound **12**.*

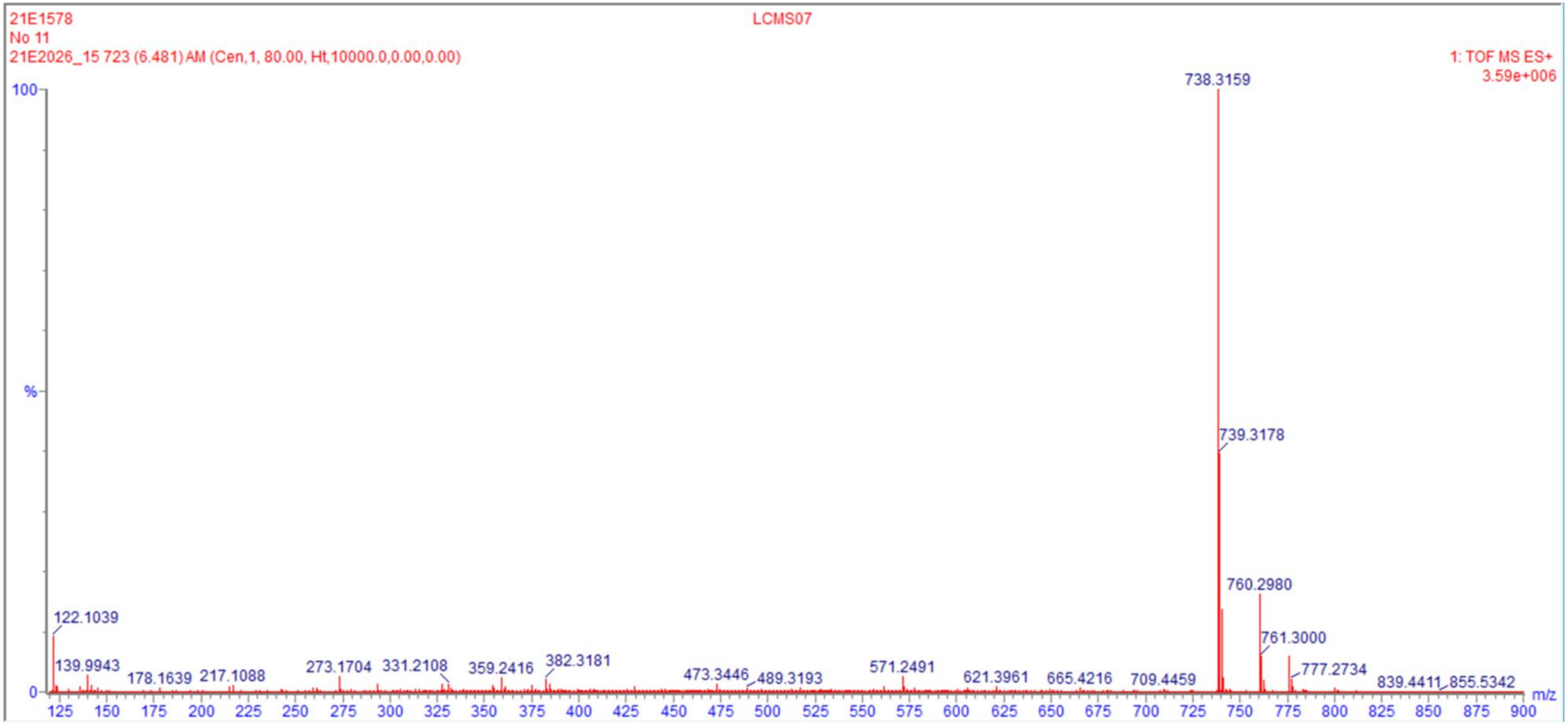

*Figure S54. ESI-TOF mass spectrum of compound **13**.*

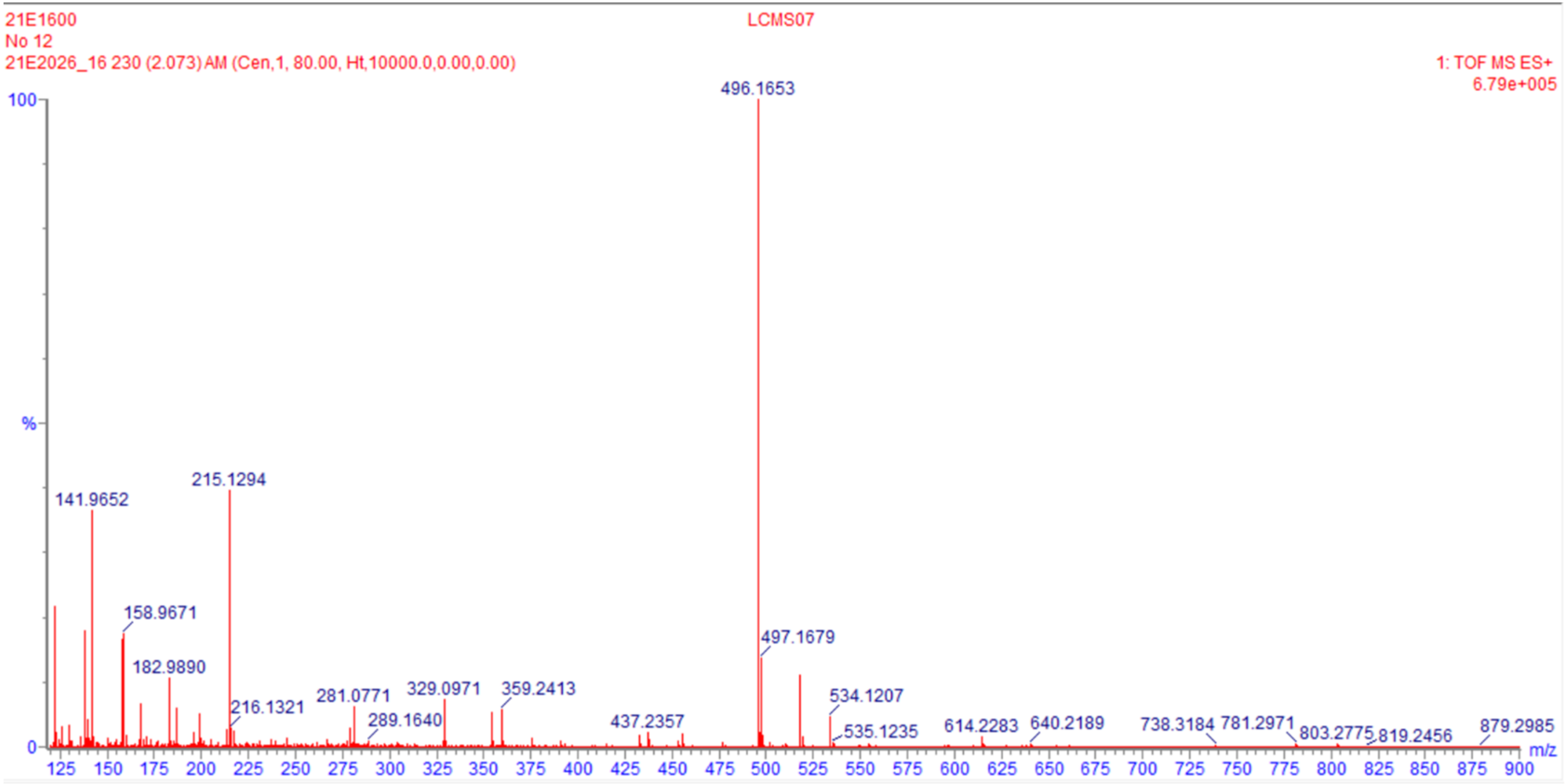

*Figure S55. ESI-TOF mass spectrum of compound **14**.*

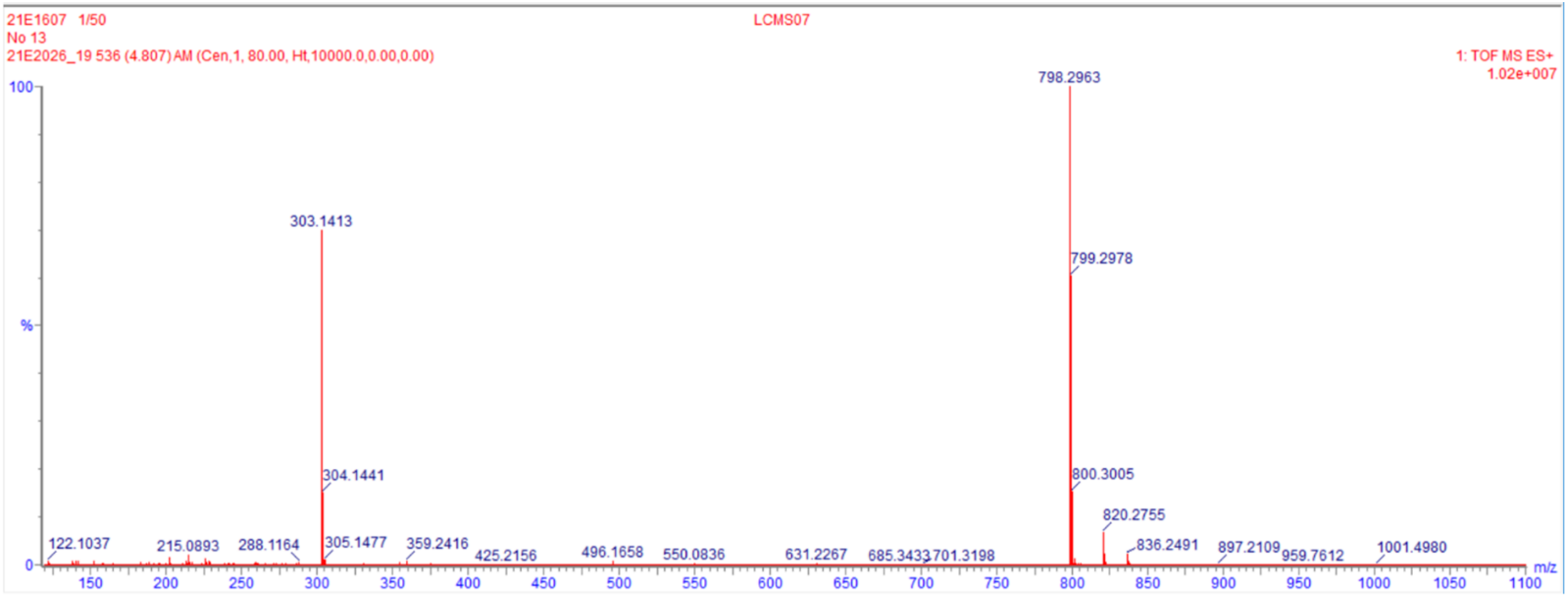

*Figure S56. ESI-TOF mass spectrum of compound **15**. M/z 303 is a fragment of DMT.*

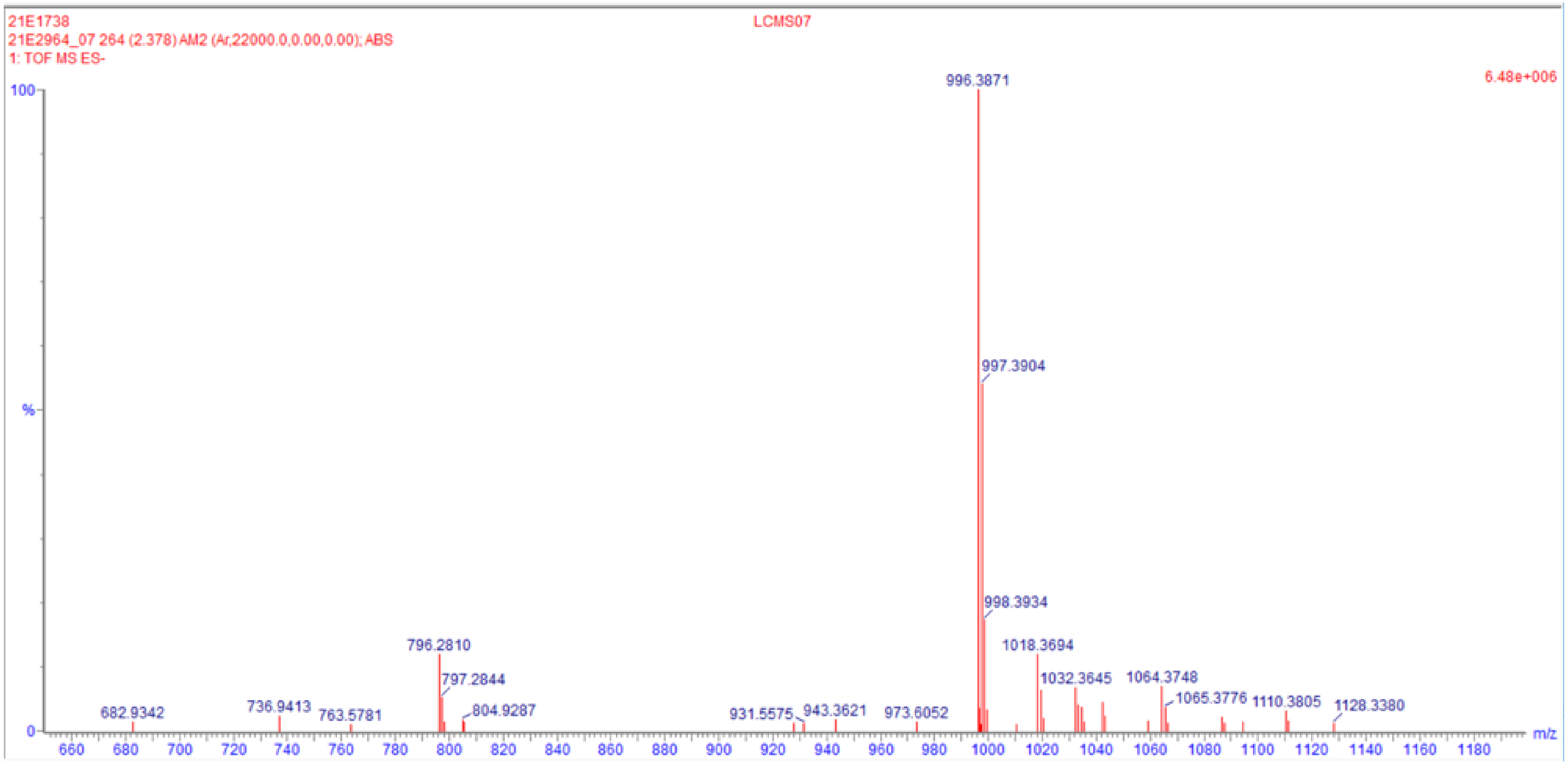

Figure S57. ESI-TOF mass spectrum of compound ***16.***
